# PffBT4T-2OD Based Solar Cells with Aryl-Substituted *N*-Methyl-Fulleropyrrolidine Acceptors

**DOI:** 10.3390/ma12244100

**Published:** 2019-12-08

**Authors:** Hugo Gaspar, Flávio Figueira, Karol Strutyński, Manuel Melle-Franco, Dzmitry Ivanou, João P. C. Tomé, Carlos M. Pereira, Luiz Pereira, Adélio Mendes, Júlio C. Viana, Gabriel Bernardo

**Affiliations:** 1 IPC/i3N—Institute for Polymers and Composites, University of Minho, Campus de Azurém, 4800-058 Guimarães, Portugal; hugo.da.silva.gaspar@gmail.com (H.G.); jcv@dep.uminho.pt (J.C.V.); 2 QOPNA & LAQV-REQUIMTE, Department of Chemistry, University of Aveiro, 3810-193 Aveiro, Portugal; jtome@ua.pt; 3 CICECO—Aveiro Institute of Materials, Department of Chemistry, University of Aveiro, 3810-193 Aveiro, Portugal; strutynski.karol@gmail.com (K.S.); manuelmelle.research@gmail.com (M.M.-F.); 4 LEPABE, Department of Chemical Engineering, University of Porto, 4200-465 Porto, Portugal; ivanou@fe.up.pt (D.I.); mendes@fe.up.pt (A.M.); 5 CQE and Departamento de Engenharia Química, Instituto Superior Técnico, Universidade de Lisboa, Av. Rovisco Pais, 1049-001 Lisboa, Portugal; 6 CIQUP, Department of Chemistry and Biochemistry, University of Porto, Rua do Campo Alegre, w/n, 4169-007 Porto, Portugal; cmpereir@fc.up.pt; 7 Department of Physics and i3N—Institute for Nanostructures, Nanomodelling and Nanofabrication, University of Aveiro, 3810-193 Aveiro, Portugal; luiz@ua.pt

**Keywords:** organic photovoltaics, fullerene derivatives, electron acceptors, fulleropyrrolidine acceptors, regioisomers of C_70_ mono-adducts

## Abstract

Novel C_60_ and C_70_ *N*-methyl-fulleropyrrolidine derivatives, containing both electron withdrawing and electron donating substituent groups, were synthesized by the well-known Prato reaction. The corresponding highest occupied molecular orbital (HOMO)/lowest unoccupied molecular orbital (LUMO) energy levels were determined by cyclic voltammetry, from the onset oxidation and reduction potentials, respectively. Some of the novel fullerenes have higher LUMO levels than the standards PC_61_BM and PC_71_BM. When tested in PffBT4T-2OD based polymer solar cells, with the standard architecture ITO/PEDOT:PSS/Active-Layer/Ca/Al, these fullerenes do not bring about any efficiency improvements compared to the standard PC_71_BM system, however they show how the electronic nature of the different substituents strongly affects the efficiency of the corresponding organic photovoltaic (OPV) devices. The functionalization of C_70_ yields a mixture of regioisomers and density functional theory (DFT) calculations show that these have systematically different electronic properties. This electronic inhomogeneity is likely responsible for the lower performance observed in devices containing C_70_ derivatives. These results help to understand how new fullerene acceptors can affect the performance of OPV devices.

## 1. Introduction

Standard inorganic solar cells can achieve high efficiencies, but they possess some disadvantages including an elaborated and costly production. To overcome these drawbacks many efforts were made to develop several third-generation thin-film solar technologies. As an example, organic photovoltaic cells (OPVs) [1,2,3,4,5] can be low-cost production since they can be manufactured in larger areas on flexible and lightweight plastic substrates via solution and printed using high-throughput roll-to-roll methods (R2R) [6]. In recent years, the OPVs have experienced significant developments in power conversion efficiency (PCE), attaining recently over 16% for single-junction devices [7,8,9] and PCE over 17% for tandem cells [10].

The small band gap donor polymer PffBT4T-2OD, poly [(5,6-difluoro-2,1,3-benzothiadiazol-4,7-diyl)-alt-(3,3′″-di(2-octyldodecyl)-2,2′; 5′, 2″; 5″,2′″-quaterthio phen-5,5′″-diyl)], also commonly known as PCE11, has been attracting large interest due to its potential to fabricate high performing OPV devices [11,12,13,14,15,16]. Its high crystallinity and relatively high SCLC hole mobility (1.5–3.0 × 10^−2^ cm^2^ V^−1^ s^−1^), allow its good performance in an OPV device, when used in relatively thick (~300 nm) bulk-heterojunction (BHJ) layers with higher light absorption capabilities. PffBT4T-2OD exhibits a strong tendency to aggregate in solution [13] characterized by the formation of a gel at room temperature. Therefore, PffBT4T-2OD based devices are usually cast from warm solutions (>60 °C), which then aggregate or crystallize during cooling and film-forming processes.

The BHJ layer plays a key role in the organic-based photovoltaic system, being a mixture of a p-type small bandgap conjugated donor polymer and an n-type acceptor to form a bicontinuous interpenetrating network [17]. The most common n-type materials used and developed for OPVs devices are the fullerene based-acceptors [15], in which their functionalization provides an opportunity of inserting a wide variety of different electron donor and withdrawing groups with direct influence on the location of the HOMO-LUMO levels and the optical absorption [18,19]. The two most widely used electron-accepting materials in OPVs are PC_61_BM ([6, 6]-Phenyl-C_61_-butyric acid methyl ester) [20,21] and its analogue PC_71_BM ([6, 6]-Phenyl-C_71_-butyric acid methyl ester) [22], both now considered as references for all kind of fullerene acceptors, due to their good solubility, high electron mobility, and high chemical stability. A key advantage of PC_71_BM in comparison with PC_61_BM is the ellipsoidal shape of the first, as compared to the more spherical C_60_ molecule [23], providing a lower symmetry and a more extended conjugation. This enables energetic transitions that are forbidden in C_60_, resulting in an extended photo-absorption profile in the visible region of the solar spectrum [24]. Consequently, there is an increased photon harvesting and a potentially higher photocurrent for devices using PC_71_BM in detriment of PC_61_BM. For that reason, most of the studies in the last years were made for OPV devices based on PffBT4T-2OD polymer with PC_71_BM.

The effect of different processing parameters (solution temperature, concentration, spin-rate, solvent quality, and polymer *M_w_*) on the morphology and performance of PffBT4T-2OD:PC_71_BM devices was studied by Ma et al. [12]. The adjustment of solution temperature and spin rate allows the tuning of the molecular orientation and packing during the spin-coating of the BHJ. A low solution temperature and low spinning rate induce highly ordered face-on polymer packing and a high solution temperature and a high spinning rate produce poorly ordered edge-on polymer packing. The best device performance (average PCE of 10.3%) was achieved using films spun-cast at 800 rpm from a solution at 100 °C, creating a smooth film containing sufficient aggregates to yield a favourable morphology.

Zhao et al. [25] studied the impact of four different additives (1,8-octanedithiol, 1,8-diiodooctane, diphenylether, and chloronaphthalene) on the performance of PffBT4T-2OD:PC_71_BM devices. Chloronaphthalene produced the best devices (PCE = 10.23%), with enhanced polymer crystallinity in the (100) direction corresponding to the alkyl stacking peak located at a *q* value of 0.29 Å^−1^, which facilitates charge transport within the BHJ.

The effect of different isomers of PC_71_BM on the photovoltaic properties of PffBT4T-2OD:PC_71_BM blend films was investigated by Umeyama et al. [16]. It should be noted that the PC_71_BM used in OPV devices normally consists of a mixture of α- and β-type isomers (in approximately 80–90% and 10–20%, respectively). The α-isomer is composed of two enantiomers, while the β-isomer consists of two diastereomers in which the phenyl group is extended toward opposite directions [26]. Interestingly, the authors found that one of the diastereomers of the β-isomer (isomer β1) shows an extraordinary cohesion in the blended film, deteriorating the OPV device performance. In fact, the PffBT4T-2OD devices based on β1-PC_71_BM exhibited an extremely low PCE of 0.43%. By contrast, OPV devices using the remaining pure isomers exhibited much higher PCE, namely α-PC_71_BM (8.80%), β2-PC_71_BM (8.75%) and these slightly surpass the device with using the normal PC_71_BM isomer mixture (8.46%) [16]. The authors concluded that decreasing the amount of diastereomers of β1-PC_71_BM with high aggregation tendency improves the photovoltaic performances [16].

Although PC_71_BM is the most commonly used fullerene in organic solar cells, several other modified fullerenes are found in the literature with specific characteristics and have been assessed as acceptors in BHJs.

Liu et al. [13] performed an extensive polymer:fullerene combination, testing the use of a large number of different fullerene acceptors (PC_71_BM, PC_61_BM, TC_71_BM, PC_61_PM, TC_61_PM, and ICMA) in devices based on the polymer PffBT4T-2OD and on other family-related polymers. The highest efficiency devices with an average PCE of 10.3% were obtained with the fullerene TC_71_BM.

More recently, Zhang et al. [15] studied the effect of three different fullerene acceptors (PC_71_BM, PC_61_BM, and ICBA) on the performance of solar cells based on PffBT4T-2OD. The investigation showed that despite PffBT4T-2OD:ICBA devices had a much higher *V_OC_* (0.94 V) than the corresponding devices using PC_71_BM and PC_61_BM (0.77 V, 0.76 V, respectively), their efficiency (average PCE of 2.78%) was much lower than the efficiency of corresponding devices based on PC_71_BM and PC_61_BM (average PCE of 8.93% and 8.15% respectively). Morphological characterization allowed to elucidate that although the size of phase domains is very similar in the three different BHJs, the fullerene aggregates in the ICBA-based films have a reduced degree of order. The high LUMO level of ICBA of the corresponding BHJs are an indicator of lower initial exciton dissociation and associated with the reduced ordering within the ICBA domains results in increased geminate recombination of the photogenerated electrons in the fullerene-rich domains and a consequently reduced PCE of the corresponding devices.

Fullerene derivatives display a wide range of physical and chemical properties that make them attractive for the preparation of supramolecular assemblies and for organic photovoltaics [27,28,29]. For instance, attaching the proper organic addends on fullerenes, it is possible to tune their solubility, energy levels, molecular interactions, surface energy, orientation in the solid-state as well as electron mobility [5,30]. However, systematic studies testing newly synthesized fullerenes in low bandgap polymers such as PffBT4T-2OD, are still scarce in the literature.

In this work we synthesize some new C_60_-based and C_70_-based fullerenes, bearing electron-donating and electron-withdrawing functionalities, and we test their effect as electron acceptors in the figures of merit of polymer solar cells based on the polymer PffBT4T-2OD. Although the PCE results are not among the best found in literature, the complete characterization of the materials and respective devices can help to further understand the role of the different fullerene acceptor structures in the performance of OPVs.

## 2. Materials and Methods

### 2.1. Materials

The starting materials used for the synthesis of novel fullerene derivatives were purchased from Solenne BV, namely: C_60_ (˃99.5% purity) with *M_w_* = 720.64 g mol^−1^ and C_70_ (˃99% purity) with *M_w_* = 840.77 g mol^−1^. The reference fullerene PC_61_BM was also purchased from Solenne BV.

The following materials, used in device fabrication, were purchased from Ossila Ltd. (Sheffield UK): (i) Poly(3,4-ethylenedioxy-thiophene):poly(styrene sulfonic acid) (PEDOT:PSS, Heraeus Clevios AI4083); (ii) the polymer PffBT4T-2OD (M302) with *M_n_* = 83,008 g.mol^−1^ and *M_w_* = 172,033 g mol^−1^ and (iii) the reference fullerene PC_71_BM (M114), purity > 99%, with empirical formula C_82_H_14_O_2_ and *M_w_* = 1030.99 g mol^−1^. The solvent used in the device fabrication was *o*-dichlorobenzene (DCB) with high purity grade and was purchased from Sigma-Aldrich. All these materials and solvent were used as received without further purification.

### 2.2. NMR Spectroscopy

^1^H and ^13^C solution NMR spectra of the functionalized fullerenes were recorded on a Bruker Avance 300 or 500 (300 or 500.13 MHz for ^1^H and 125.76 MHz for ^13^C) spectrometers. CS_2_, deuterated acetone and deuterated chloroform (99.6%, TCI Chemicals) were used as solvents and tetramethylsilane (TMS) as internal reference. The chemical shifts are expressed in (ppm).

### 2.3. Preparation of Compounds ***60a***, ***60b***, ***60c***, ***70a***, ***70b*** and ***70c***

A mixture of C_60_ (100 mg, 0.14 mmol, 1 eq.) or C_70_ (100 mg, 0.12 mmol), *N*-methylglycine (0.35 mmol) and the corresponding aldehyde (0.70 mmol) in a toluene solution (100 mL) was stirred at reflux temperature for 8 h and then the solvent was removed under vacuum. The solid residue was purified by flash column chromatography (eluent: toluene/hexanes 1:3 with increasing amounts of toluene until purification of the first brown band) affording roughly 30–40% of the *N*-methyl-3,4-fulleropyrrolidine derivatives **60a**, **60b**, **60c**, **70a**, **70b,** and **70c**.

**60a:**^1^H NMR (300 MHz, CS_2_ + Acetone-d6): δ 7.81 (d, *J* = 7.3 Hz, 2H), 7.43 (td, *J* = 7.3, 1.3 Hz, 2H), 7.39–7.30 (m, 1H), 5.04 (d, *J* = 9.4 Hz, 1H), 5.00 (s, 1H), 4.34 (d, *J* = 9.4 Hz, 1H), 2.86 (s, 3H); ^13^C NMR (126 MHz, CS_2_ + Acetone-d6): δ 156.96, 154.05, 154.01, 147.75, 147.33, 147.07, 146.78, 146.73, 146.69, 146.66, 146.59, 146.56, 146.41, 146.28, 146.08, 146.04, 146.01, 145.86, 145.77, 145.70, 145.66, 145.62, 145.21, 145.14, 144.90, 143.63, 143.50, 143.17, 143.09, 143.07, 143.05, 142.80, 142.67, 142.61, 142.29, 142.19, 140.64, 139.95, 137.46, 137.38, 137.10, 136.45, 136.32, 129.34, 129.08, 83.94, 77.73, 70.41, 69.55, 40.30. (Figures S1–S3 in the Supplementary Materials)

**60b:**^1^H NMR (300 MHz, CS_2_ + Acetone-d6): δ 7.69 (d, *J* = 8.1 Hz, 2H), 6.92 (m, 2H), 5.00 (d, *J* = 9.3 Hz, 1H), 4.92 (s, 1H), 4.30 (d, *J* = 9.3 Hz, 1H), 3.81 (s, 3H), 2.82 (s, 3H); ^13^C NMR (126 MHz, Acetone-*d*_6_): δ 157.00, 154.08, 154.05, 147.78, 147.36, 147.10, 146.82, 146.76, 146.73, 146.69, 146.63, 146.60, 146.44, 146.32, 146.12, 146.08, 146.04, 145.89, 145.81, 145.74, 145.69, 145.65, 145.25, 145.18, 144.93, 143.66, 143.54, 143.20, 143.13, 143.10, 143.08, 142.84, 142.71, 142.65, 142.58, 142.48, 142.32, 142.23, 140.73, 140.68, 139.99, 137.50, 137.42, 137.14, 136.48, 136.36, 129.38, 129.11, 83.98, 77.76, 70.44, 69.58, 40.33. (Figures S4–S6)

**60c:**^1^H NMR (300 MHz, CS_2_ + Acetone-d6): δ 7.70 (d, *J* = 8.104 Hz, 2H), 6.93 (d, *J* = 8.90 Hz, 2H), 5.01 (d, *J* = 9.3 Hz, 1H), 4.93 (s, 1H), 4.30 (d, *J* = 9.3 Hz, 1H), 3.81 (s, 3H), 2.83 (s, 3H); ^13^C NMR (126 MHz, CS_2_ + Acetone-d6): δ 165.72, 156.56, 154.30, 153.47, 153.22, 147.72, 147.00, 146.88, 146.76, 146.70, 146.66, 146.57, 146.38, 146.32, 146.19, 146.05, 145.95, 145.91, 145.84, 145.76, 145.68, 145.61, 145.17, 145.07, 144.86, 143.60, 143.48, 143.15, 143.07, 143.05, 143.01, 142.74, 142.63, 142.58, 142.54, 142.51, 142.49, 142.44, 142.33, 142.27, 142.16, 142.03, 140.69, 140.42, 139.96, 136.96, 136.51, 136.21, 130.95, 130.50, 83.42, 77.42, 70.39, 69.50, 52.09, 40.32. (Figures S7–S9)

**70a:**^1^H NMR (500 MHz, CS_2_ + Acetone-*d*_6_): δ 7.95–7.05 (m, 5H), 5.50–4.29 (m, 1H), 4.30–3.92 (m, 1H), 3.71–3.27 (m, 1H), 2.61–2.43 (m, 3H); ^13^C NMR (126 MHz, CS_2_ + Acetone-*d*_6_): δ 159.43, 159.06, 158.72, 156.94, 156.61, 156.25, 155.48, 155.41, 153.94, 153.21, 153.12, 152.13, 151.93, 151.90, 151.87, 151.82, 151.75, 151.63, 151.49, 151.32, 151.24, 151.16, 151.07, 150.93, 150.70, 150.55, 150.35, 150.20, 150.16, 150.08, 149.98, 149.92, 149.87, 149.79, 149.69, 149.64, 149.55, 149.50, 149.45, 149.37, 149.26, 149.10, 148.78, 148.45, 148.22, 148.00, 147.94, 147.87, 147.71, 147.51, 147.34, 147.24, 147.10, 147.00, 146.77, 146.69, 146.67, 146.28, 146.19, 146.17, 146.09, 146.04, 145.77, 145.40, 145.29, 145.12, 144.33, 143.96, 143.90, 143.69, 143.62, 143.58, 143.52, 143.34, 143.29, 143.15, 142.81, 142.28, 142.20, 141.58, 141.38, 141.26, 141.13, 140.73, 140.67, 138.30, 138.23, 138.17, 137.87, 137.43, 136.99, 134.28, 134.07, 134.00, 133.47, 132.78, 132.52, 132.47, 131.96, 131.69, 131.65, 130.94, 130.18, 129.60, 129.25, 129.18, 129.08, 129.01, 128.98, 128.82, 127.26, 127.08, 83.72, 83.41, 82.77, 80.61, 71.34, 70.59, 70.38, 69.10, 68.55, 67.08, 66.63, 62.56, 60.81, 59.22, 58.84, 39.79, 39.77, 39.74, 39.70. (Figures S10–S12)

**70b:**^1^H NMR (500 MHz, CS_2_ + Acetone-*d*_6_): δ 8.47–7.12 (m, 4H), 4.81–4.31 (m, 1H), 4.32–4.08 (m, 1H), 4.00–3.78 (m, 3H), 3.72–3.26 (m, 1H), 2.66–2.41 (m, 3H); ^13^C NMR (126 MHz, Acetone-d6): δ 165.81, 165.69, 165.52, 165.49, 159.18, 158.77, 158.18, 156.75, 156.38, 155.53, 155.24, 155.12, 155.00, 153.74, 153.72, 152.82, 152.70, 152.38, 152.07, 151.85, 151.80, 151.77, 151.73, 151.67, 151.64, 151.56, 151.50, 151.45, 151.28, 151.18, 151.10, 151.08, 151.01, 150.98, 150.95, 150.86, 150.78, 150.64, 150.49, 150.46, 150.31, 150.29, 150.26, 150.19, 150.13, 150.10, 150.08, 150.02, 149.95, 149.82, 149.72, 149.65, 149.63, 149.62, 149.60, 149.52, 149.47, 149.44, 149.41, 149.37, 149.33, 149.30, 149.26, 149.20, 149.17, 149.09, 149.03, 148.74, 148.68, 148.43, 148.41, 148.38, 148.33, 148.17, 148.06, 147.93, 147.85, 147.81, 147.79, 147.73, 147.60, 147.55, 147.51, 147.48, 147.43, 147.41, 147.38, 147.30, 147.27, 147.22, 147.18, 147.14, 147.05, 147.01, 146.95, 146.92, 146.88, 146.84, 146.81, 146.69, 146.64, 146.60, 146.58, 146.56, 146.54, 146.31, 146.22, 146.18, 146.16, 146.08, 145.93, 145.79, 145.75, 145.74, 145.72, 145.71, 145.33, 144.99, 144.95, 144.73, 144.64, 144.33, 144.31, 143.92, 143.89, 143.82, 143.80, 143.68, 143.66, 143.65, 143.59, 143.58, 143.45, 143.29, 143.26, 143.23, 143.22, 143.14, 143.13, 142.42, 142.38, 142.04, 141.96, 141.49, 141.41, 141.31, 141.10, 141.03, 140.97, 140.86, 140.71, 140.65, 140.53, 138.43, 138.08, 137.91, 137.73, 134.23, 134.21, 134.15, 134.00, 133.90, 133.36, 132.71, 132.69, 132.46, 132.40, 132.04, 132.02, 131.95, 131.88, 131.69, 131.62, 131.58, 131.49, 131.10, 130.92, 130.83, 130.75, 130.64, 130.35, 130.22, 130.14, 130.00, 129.58, 127.13, 127.06, 126.02, 83.23, 82.85, 82.24, 80.09, 71.07, 70.53, 70.17, 69.05, 68.52, 66.90, 66.68, 66.59, 62.52, 60.79, 59.19, 58.83, 52.19, 52.11, 52.04, 52.00, 39.80, 39.79, 39.76, 39.72. (Figures S13–S15)

**70c:**^1^H NMR (500 MHz, CS_2_ + Acetone-*d*_6_): δ 7.78–6.64 (m, 4H), 4.78–4.22 (m, 1H), 4.23–3.99 (m, 1H), 3.99–3.69 (m, 3H), 3.65–3.17 (m, 1H), 2.60–2.35 (m, 3H); ^13^C NMR (126 MHz, CS_2_ + Acetone-d6): δ 160.41, 160.24, 160.11, 159.95, 159.60, 159.28, 159.07, 157.08, 156.71, 156.65, 155.78, 155.53, 154.10, 154.07, 153.55, 153.31, 152.75, 152.19, 152.01, 151.95, 151.93, 151.84, 151.81, 151.78, 151.66, 151.56, 151.37, 151.26, 151.18, 151.15, 151.03, 150.78, 150.76, 150.54, 150.43, 150.37, 150.31, 150.23, 150.15, 150.08, 149.92, 149.90, 149.86, 149.80, 149.75, 149.63, 149.60, 149.57, 149.53, 149.50, 149.43, 149.33, 149.24, 149.18, 148.82, 148.78, 148.55, 148.51, 148.44, 148.21, 148.07, 148.03, 148.00, 147.95, 147.83, 147.79, 147.68, 147.64, 147.60, 147.58, 147.54, 147.44, 147.38, 147.35, 147.31, 147.22, 147.16, 147.10, 147.07, 146.86, 146.79, 146.76, 146.69, 146.62, 146.38, 146.34, 146.29, 146.26, 146.21, 146.15, 145.83, 145.68, 145.65, 145.47, 145.17, 144.91, 144.78, 144.38, 144.03, 143.96, 143.94, 143.88, 143.82, 143.74, 143.70, 143.66, 143.58, 143.42, 143.33, 143.21, 143.18, 143.09, 142.53, 142.42, 142.20, 141.67, 141.47, 141.44, 141.38, 141.23, 141.06, 141.00, 140.82, 140.78, 138.35, 138.31, 137.98, 137.83, 134.35, 134.14, 134.06, 133.66, 132.85, 132.53, 132.16, 132.14, 132.05, 131.93, 131.73, 131.68, 131.63, 131.26, 130.92, 130.00, 129.26, 128.83, 128.80, 126.30, 126.25, 114.99, 114.46, 87.54, 83.31, 83.05, 82.42, 80.22, 73.64, 71.61, 70.65, 70.56, 69.04, 68.50, 67.01, 66.59, 62.56, 60.81, 59.22, 58.82, 55.47, 55.36, 55.26, 55.22, 40.07, 39.76, 39.72, 39.69, 39.66. (Figures S16–S18)

### 2.4. Preparation of Compounds ***60d*** and ***70d***

A toluene solution (100 mL) containing C_60_ (100 mg, 0.14 mmol) or C_70_, (0.12 mmol), *N*-methylglycine (0.35 mmol) and the corresponding aldehyde (0.70 mmol) was stirred until reflux temperature. After this, 0.67 mmol of the corresponding aldehyde was added to the reaction every three hours (3 times) and the reaction was maintained for another 15 h at reflux temperature. The reaction mixture was then concentrated and purified by flash column chromatography, using toluene/hexanes (1:3) as eluent, with increasing amounts of toluene until purification of the first brown band, affording roughly 30–40% of the *N*-methyl-3,4-fulleropyrrolidine derivatives **60d** and **70d**.

**60d:**^1^H NMR (300 MHz, CS_2_ + Chloroform-d): δ 5.48 (s, 1H), 5.02 (d, *J* = 9.39 Hz, 1H), 4.23 (dd, *J* = 9.46, 3.109 Hz, 1H), 2.89 (d, *J* = 2.439 Hz, 3H); ^13^C NMR (126 MHz, CS_2_ + Chloroform-d): δ 156.69, 154.29, 153.24, 152.14, 148.01, 147.97, 147.01, 146.98, 146.90, 146.87, 146.77, 146.75, 146.64, 146.47, 146.40, 146.33, 146.29, 146.23, 146.20, 146.14, 146.03, 145.94, 145.88, 145.43, 145.17, 143.78, 143.72, 143.38, 143.29, 143.27, 142.96, 142.88, 142.79, 142.67, 142.63, 142.58, 142.39, 142.33, 140.92, 140.81, 140.27, 137.25, 136.71, 136.33, 76.50, 75.22, 70.20, 70.01, 54.87, 40.23. (Figures S19–S21)

**70d:**^1^H NMR (500 MHz, Chloroform-*d*): δ 4.79–4.48 (m, 1H), 4.38–3.67 (m, 1H), 3.58–3.09 (m, 1H), 2.65–2.38 (m, 3H); ^13^C NMR (126 MHz, Chloroform-*d*): δ 158.58, 158.24, 157.32, 156.32, 155.23, 155.20, 154.91, 154.81, 153.54, 153.23, 153.12, 152.24, 151.88, 151.63, 151.53, 151.51, 151.49, 151.32, 151.21, 151.16, 151.00, 150.94, 150.86, 150.83, 150.76, 150.44, 150.39, 150.03, 149.98, 149.68, 149.62, 149.57, 149.50, 149.48, 149.47, 149.34, 149.27, 149.17, 148.93, 148.79, 148.41, 148.20, 147.75, 147.58, 147.53, 147.50, 147.45, 147.35, 147.27, 147.21, 147.18, 147.15, 147.10, 147.00, 146.85, 146.80, 146.75, 146.67, 146.37, 146.32, 146.25, 146.13, 146.09, 145.94, 145.90, 145.80, 145.54, 145.37, 145.05, 144.84, 144.58, 144.51, 144.10, 143.61, 143.53, 143.44, 143.39, 143.21, 143.05, 143.00, 142.97, 142.88, 142.14, 141.69, 141.01, 140.69, 140.56, 140.37, 140.31, 140.14, 140.03, 139.29, 137.61, 137.45, 137.21, 134.02, 133.82, 133.76, 133.71, 133.36, 132.25, 132.15, 131.77, 131.60, 131.42, 131.34, 74.88, 71.71, 69.83, 69.64, 68.88, 68.60, 67.99, 65.89, 65.75, 65.55, 62.53, 60.88, 58.80, 39.82. (Figures S22–S24)

### 2.5. Cyclic Voltammetry

Autolat PGSTAT302N potentiostat was used in electrochemical experiments. Voltammograms were recorded using a three-electrode cell arrangement; a polished glassy-carbon (GC) pin (3 mm in diameter) served as a working electrode, a platinum wire as a counter electrode, and a nonaqueous Ag|Ag^+^ reference electrode with an internal solution of AgNO_3_ (0.01 M) and 0.1 M of Bu_4_NPF_6_ in acetonitrile. The fullerenes (ca. 0.6 mg/mL) were dissolved in a solvent mixture of 4:1 (by volume) chlorobenzene:acetonitrile with the addition of 0.1 M Bu_4_NPF_6_ as a supporting electrolyte. Before measurements, the solutions were deaerated by purging high-purity Argon for 7 min. Cyclic voltammograms were recorded at a potential scan rate of 100 mV/s. During the measurements, the Argon flow was kept above the solution in the cell. All electrode potentials were quoted with respect to equilibrium potential (*E_1/2_*) of Fc^+^/Fc redox couple in the same solvent mixture; *E_1/2_* (Fc^+^/Fc) = 0.29 V vs. Ag|Ag^+^. The LUMO and HOMO energy levels were estimated from the onset potential of the reduction ($E_{Red}^{on}$) and oxidation ($E_{Ox}^{on}$) respectively: *E_LUMO_* = −4.9 − $E_{Red}^{on}$; *E_HOMO_* = −4.9 − $E_{Ox}^{on}$.

### 2.6. Ab Initio DFT Calculations

Density Functional Theory calculations at the PBEh-3c level were performed to derive all molecular structures [31]. To compare with voltammetry experiments, PBE-def2-TZVP level calculations were used as they show larger accuracy than hybrid functionals [32,33,34]. For C_70_ systems both isomers, α and β, were computed. For simplicity, only one diasteroisomer for each isomer was considered after preliminary calculations showed similar results for both diasterisomeric forms. All calculations were performed with the ORCA 4.2 program [35].

### 2.7. Absorption Spectroscopy

In the first stage, UV-Vis absorption spectroscopy was used to characterize the optical properties of the pure fullerenes in *o*-dichlorobenzene solution. Although attempts have also been made to measure the UV-Vis spectra of spin-coated thin films of the pure fullerenes on quartz windows, these were not successful due to the difficulty in preparing homogeneous thin films.

In a posterior stage, UV-Vis absorption spectroscopy was used to evaluate the effect of fullerenes in the light absorption of the different blends, which is one of the first steps to charge generation. Absorption spectra (UV-Vis) were obtained on a Shimadzu UV-2501PC spectrophotometer, in the 350–800 nm range in a solid-state film deposited in quartz substrates.

### 2.8. Device Fabrication

The OPV devices employ a standard structure ITO/HTL/Active layer/Ca/Al. PEDOT:PSS was used as a hole transport layer (HTL). ITO (Indium Tin Oxide) has a sheet resistance of 20 Ω/□. The active layers were all spin-coated from a solution of *o*-dichlorobenzene with the polymer PffBT4T-2OD and several fullerene derivatives, having concentrations of 4.0 mg·mL^−1^ and 4.8 mg·mL^−1^ respectively (1:1.2 mass ratio). At this point, it is worth mentioning that other polymer:fullerene mass ratios were initially screened (namely 1:3 and 3:1) but later discarded because the corresponding devices exhibited lower figures of merit. Although the majority of the previous works use a solvent mixture of chlorobenzene:*o*-dichlorobenzene (1:1) to dissolve the polymer, in the present work due to the relatively high *Mw* of the polymer used, it proved to be more appropriate the use of pure *o*-dichlorobenzene which has a stronger solubilizing power than the chlorobenzene/*o*-dichlorobenzene mixture. Some preliminary tests have proved no performance benefits on the use of additives and therefore no additives were used on subsequent device preparation. Due to the strong aggregation and gelation tendency of the polymer PffBT4T-2OD in solution, the active layers were spin-coated from pre-heated solutions (120 °C) at a spin speed of 800 rpm onto the PEDOT:PSS/glass substrate that was pre-heated to 120 °C. The active layer was spin-cast in a nitrogen-filled glove box. The films were then left inside the glove box for 1 h to dry. The cathode evaporation was then made sequentially composed by 5 nm Calcium (Ca) and 100 nm Aluminium (Al) on top of the active layer under a vacuum < 2 × 10^−6^ mbar to form the top electrode contact.

### 2.9. Device Performance Characterization

Photovoltaic properties of the devices were determined using a Newport-Oriel 96,000 AM 1.5 Global solar simulator which was calibrated using an NREL standard silicon solar cell to ensure an irradiance level of 1000 W/m^2^. An aperture mask was used to limit the light-exposed area of the device to 2.6 mm^2^, avoiding edge effects. All measurements were made at room temperature.

### 2.10. Morphological Characterization Using Atomic Force Microscopy (AFM)

Atomic Force Microscopy (AFM) in tapping mode was used to image the surface morphology of the PffBT4T-2OD: fullerene thin films. AFM experiments were performed using a Molecular Imaging PicoLE AFM. The measurements were performed in contact mode and several scans were imaged in flattened mode data to remove the background slope. The scan size of topographic AFM images presented in all experiments is 5 × 5 μm^2^.

## 3. Results and Discussion

**Figure 1:**
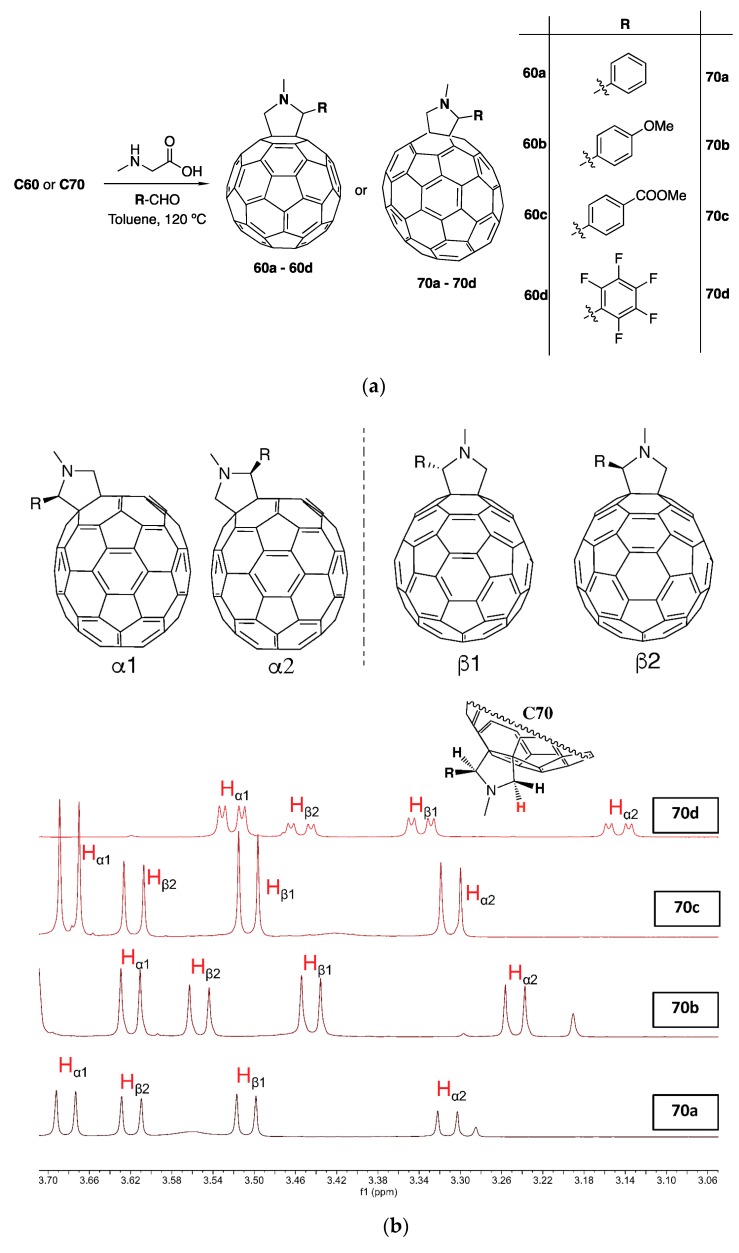
(**a**) Preparation method used in the synthesis of compounds **60a** to **60d** and **70a** to **70d**; (**b**) Structures and ^1^H NMR of the four different isomers (α1, α2, β1, β2) present in each of the C_70_ derivatives **70a** to **70d**.

In this work, we started by synthesizing mono-functionalized C_60_ and C_70_ fullerenes containing electron-donating and withdrawing groups, as shown in Figure 1a. This was achieved by the well-known Prato reaction, a controlled cycloaddition reaction in which a pyrrolidine ring is fused with a 6, 6 ring junction of both C_60_ or C_70_ [13,36]. Compounds **60a**–**60d** and **70a**–**70d** were prepared by reaction of the appropriate azomethine ylide precursors respectively with C_60_ and C_70_. The reactive 1, 3-dipoles were generated in situ and compounds **60a**–**60d** and **70a**–**70d** were synthesized according to Figure 1a, using *N*-methyl glycine (sarcosine) and the corresponding aldehyde. Due to the large variety of substituted azomethine ylides that can be generated from readily accessible starting materials, highly functionalized pyrrolidine rings in C_60_ and C_70_ can be easily obtained in moderate to good yields [36].

We observed that the solubility of the resulting C_70_ derivatives was greatly improved when compared with the solubility of the C_60_ derivatives: while derivatives **70a**–**70d [37]** were soluble in dichloromethane, derivatives **60a**–**60d** were far less soluble, and only solvents such as toluene and CS_2_ could dissolve them. The final products were separated from the unreacted starting material (C_60_ or C_70_) and other by-products by silica gel chromatography using a mixture of toluene/hexane. Most of the substituents introduced on the fullerenes provided enough polarity to easily separate the final materials from the starting materials as the second coloured band eluted from the silica gel chromatography.

The structures of **60a**–**60d** and **70a**–**70d** derivatives were all confirmed by ^1^H NMR spectroscopy (Figures S1–S24 in Supplementary Materials). However, it is important to note that the ^1^H NMR spectra of the C_70_ derivatives display four signals attributed to the pyrrolidine protons as a characteristic feature of four distinct C_70_ isomeric products. Indeed, unlike C_60_, in which all carbon atoms and double bonds are initially equivalent, the lower symmetry of C_70_ gives rise to a mixture of four isomers. As shown in Figure 1b, two sets of [6, 6] mono-adducts are expected and the integration based on one of these protons (^1^H, Figure 1b) allowed us to estimate the fractions of each isomer in the C_70_ derivatives **70a** to **70d**. The singlet signal of the ^1^H proton is clearly split into four signals, corresponding to the α1, α2, β1, and β2 C_70_ mono-adducts in a ratio where the α isomers tend to increase with the electron-withdrawing effect around the phenyl substituent. The ^1^H NMR data of the pyrrolidine is summarized in Table 1. Other techniques such as HPLC can further confirm the isomeric nature of these mixtures. For instance, Urbani et al. [37] have prepared C_70_-fuleropyrrodines with a similar approach here described and confirmed the presence of two kinds of isomeric products, namely, α and the β-types. However, differentiation within the α isomers or the β isomers was not possible. The same authors also found that under Prato experimental conditions, only [6, 6] C_70_ mono-adducts were isolated and the presence of the [5, 6] isomeric products could not be detected by classical analytical methods (HPLC and NMR).

**Figure 2:**
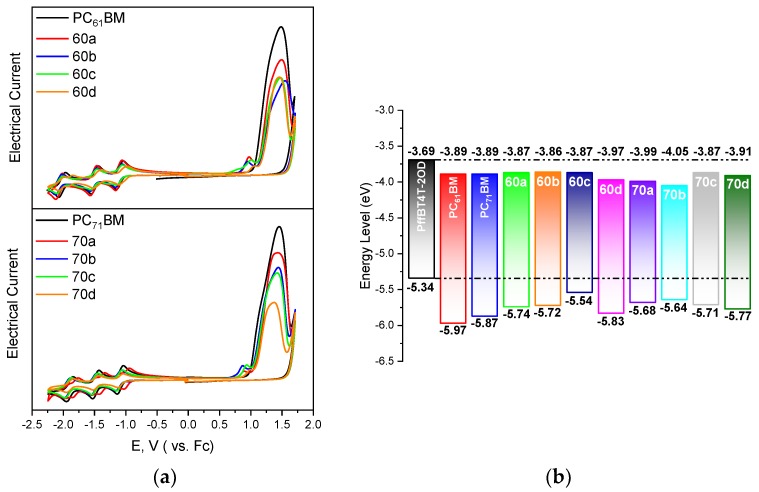
(**a**) Cyclic Voltammetry curves for all different materials. The Electrical Current scale is arbitrary shifted in order to show all curves; (**b**) Scheme of highest occupied molecular orbital (HOMO) and lowest unoccupied molecular orbital (LUMO) levels for all materials as calculated from cyclic voltammetry. The HOMO and LUMO levels for PffBT4T-2OD as indicated in [13] are also shown.

The electrochemical properties of the synthesized fullerenes were then determined using cyclic voltammetry. Figure 2a shows the Cyclic Voltammetry (CV) curves for all samples. The experimentally determined energy levels are summarized in Table S1 in Supplementary Information and represented graphically in Figure 2b. In Figure 2b, we also represent the HOMO/LUMO levels of PffBT4T-2OD as taken from the literature [13]. We note that our experimentally determined HOMO/LUMO levels for the standard fullerenes PC_61_BM and PC_71_BM are in very good agreement with literature data [38,39] which guarantees the reliability of our measurements for all the remaining fullerenes. We note that some of the novel fullerenes (**60a**, **60b**, **60c,** and **70c**) have slightly higher LUMO levels than the standards PC_71_BM and PC_61_BM, which in theory should favour higher *V_OC_* and PCE assuming that all other factors remain unchanged.

**Figure 3:**
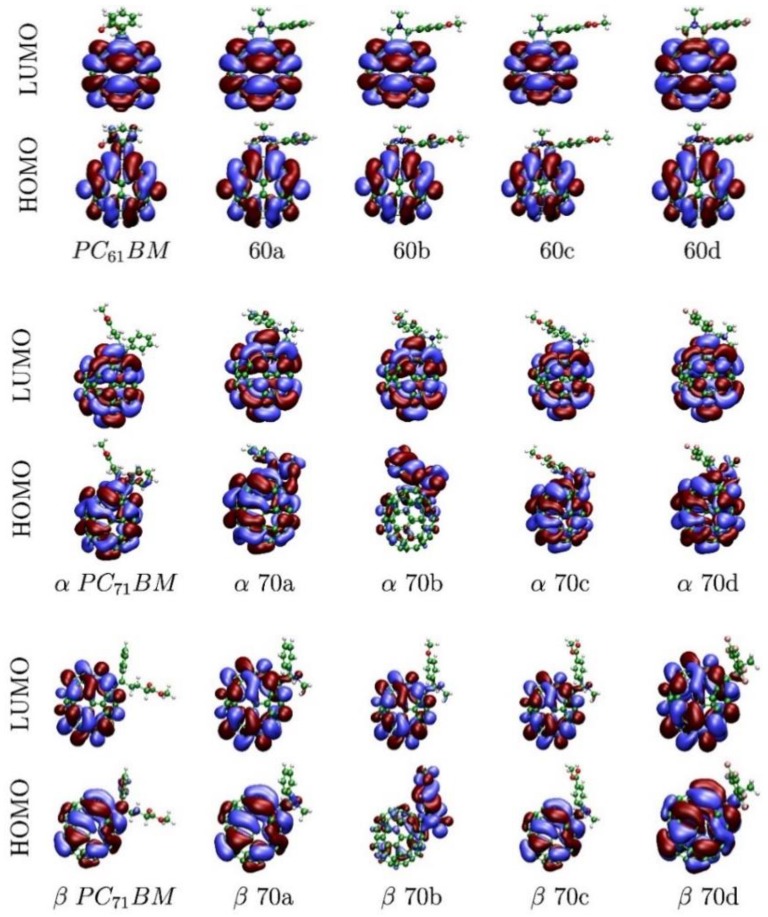
Frontier orbitals of pristine and functionalized C_60_ and C_70_ molecules.

The HOMO and LUMO energies were computed with DFT and show a reasonable agreement with the voltammetry derived values, Table 2. In detail, LUMO energies show a remarkable agreement with only **70a** and **70b** molecules showing differences larger than 0.1 eV, while HOMOs show generally larger divergences. Interestingly, when the energies of the frontier orbitals of α and β C_70_ isomers are compared, HOMOs are stabilized while LUMOs are destabilized. Consequently, all α isomers have systematically larger HOMO-LUMO gaps than β isomers. Also, for all functionalized C_60_ and some C_70_ molecules, both frontier orbitals have most electron density localized in the fullerene moieties, Figure 3. There are only two exceptions: **70b** and, to a lesser extent, **70a** where there is also density on the functional groups. This observation is related to the electron donor ability of the different R groups (Figure 1a). In fact, the computed HOMO energies of the four different groups follow the trend R_b_ > C_70_ > R_a_ > R_c_ ≃ R_d_, which accounts for the anomalous densities.

The UV-Vis absorption spectra of pure solutions of each fullerene in o-dichlorobenzene were recorded and are shown in Figure S25 in the Supplementary Information. As expected, the C_70_-based fullerenes display a broader photo-absorption profile in the visible region of the solar spectrum and this is due to the lower and more extended conjugation of the C_70_ cage that enables energetic transitions that are forbidden in C_60_ [24]. Although the main exciton creation is assumed to occur in the polymer, such increased photon harvesting potentially allows higher photocurrents for devices using C_70_-based fullerenes than for their C_60_-based analogues.

**Figure 4:**
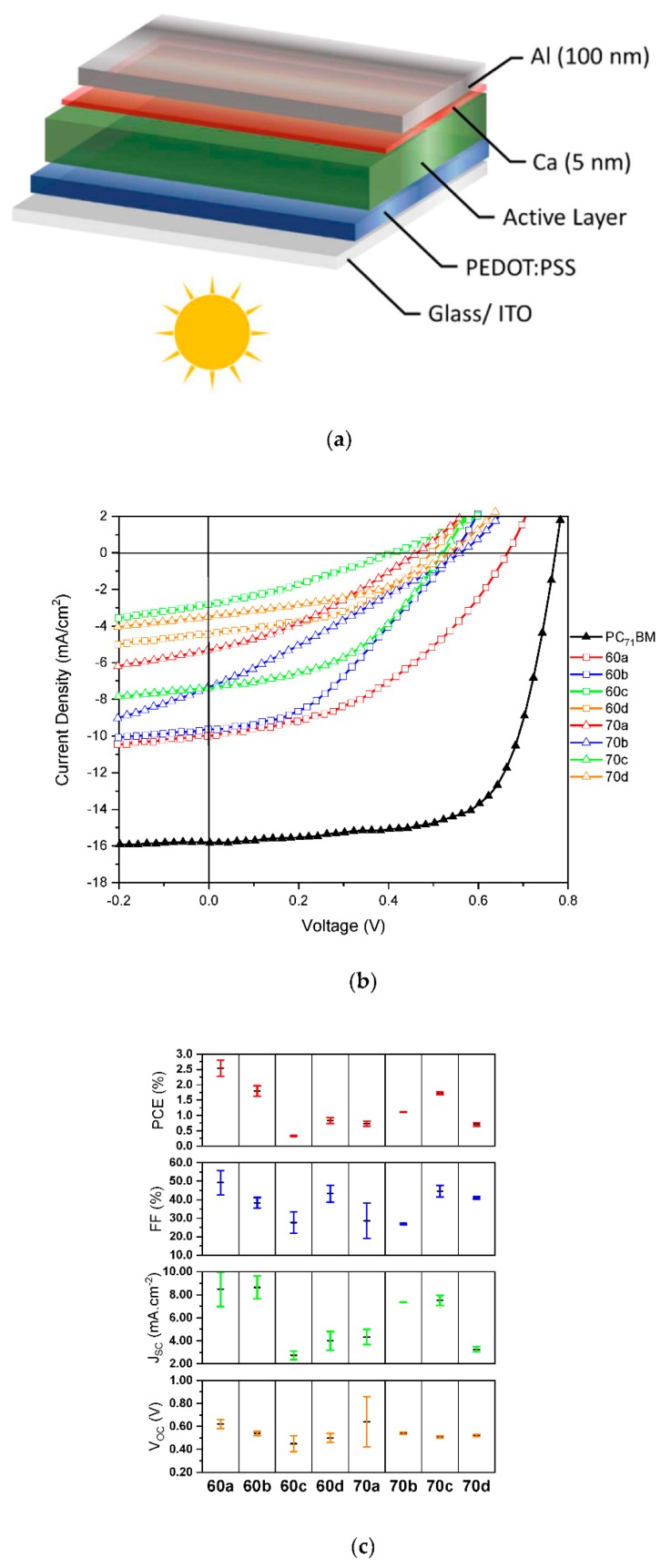
(**a**) A schematic of the standard device structure used in the fabrication of devices; (**b**) Representative electrical current density—applied voltage (J–V) curves for PffBT4T-2OD based devices with each particular type of fullerene species **60a**–**60d** and **70a**–**70d**; (**c**) Overall device metrics for PffBT4T-2OD based devices using the different fullerenes **60a**–**60d** and **70a**–**70d**.

The current density-voltage (J–V) curves of devices with standard architecture, as shown in Figure 4a, and processed with the eight different fullerenes, as well as a control device using the standard PC_71_BM, are shown in Figure 4b. The main figures of merit for the different devices are represented schematically in Figure 4c and indicated in Table 3. Although device data using the standard, less common, PC_61_BM has not been included, according to literature the PCE of PffBT4T-2OD:PC_61_BM devices in only ~10% lower than the PCE of PffBT4T-2OD:PC_71_BM devices [15]. We note that in order to guarantee the reproducibility and the reliable comparison of device data between different fabrication batches, every time a new batch of devices was made, a few devices with PC_71_BM were included as a control of the general quality of the whole device fabrication process. As shown in Figure 4b and Table 3, all the new fullerenes produced devices with considerably lower figures of merit than the reference devices with PC_71_BM, even though most of the novel fullerenes possess favorable LUMO levels.

Interestingly, we also observe that despite their higher light absorption, C_70_-based fullerenes (open triangles in Figure 4b) in general produce lower efficiencies (best PCE of 1.76% for **70c**) than the C_60_-based fullerenes (open squares—best PCEs of 2.84% for **60a** and 2.04% for **60b**). When comparing fullerenes with identical functionalization we observe that **60a**, **60b**, and **60d** produce devices with better figures of merit than respectively **70a**, **70b**, and **70d**. Only in the case of **60c** and **70c,** the opposite relationship is observed. The inferior performance that we generally observe in our C_70_-based devices, compared to C_60_-based devices, is most likely due to the energetic disorder introduced in these systems by the presence of several isomers, as our DFT calculations clearly show that the C_70_ isomers have different electronic properties. This effect has been previously observed in the case of PC_71_BM isomers, in a study where the authors concluded that one of the β-isomers present in the mixture of four isomers (α1, α2, β1, and β2) does not contribute to the efficiency of the overall mixture [16]. In particular, Umeyama and co-workers found that devices prepared using a blend of PffBT4T-2OD with the purified β1-PC_71_BM isomer exhibited an extremely low PCE of 0.43%, due to the high aggregation tendency of β1-PC_71_BM in the blend film. In our present work, this effect can be even more pronounced since the synthetic methodologies employed produced amounts of β1-isomer up to 30% [37,40]. It is worth mentioning that the effect of isomers of C_70_-based mono-adducts in OPVs has been receiving special attention [16,41,42,43,44] by the group of Prof Imahori at Kyoto University and a very recent review by this group [41] calls the attention of the research community to the great importance of this issue which has been so far largely ignored.

In Figure 4b it is noticeable the change in the shape of some J–V curves compared to the data when conventional PC_71_BM is employed in the active layer. For instance, devices with fullerene **60c** exhibit a very pronounced influence of a space charge limited current (SCLC) [45], and similar but less pronounced behaviour is also observed in the remaining fulleropyrrolidine based devices. Together with the decrease of *V_OC_* and *J_SC_* for all devices (compared to the PC_71_BM) the fill-factor *FF* also decreases due to the mentioned J-V shape. At this point it is worth noting that a large number of previous studies in the literature have reported new fullerenes that despite exhibiting enhanced LUMO levels with respect to PC_61_BM or PC_71_BM, originated devices with considerably lower PCE [46,47,48,49,50,51,52,53,54,55] and also with simultaneously lower PCE and *V_OC_* [50,51,52,53,54,55] than reference devices based on PC_61_BM or PC_71_BM.

**Figure 5:**
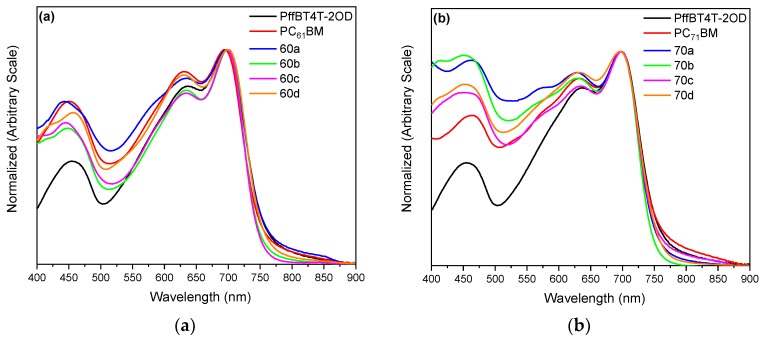
UV-Vis absorption spectra of a PffBT4T-2OD pristine film and of PffBT4T-2OD:fullerene blend films with (**a**) C_60_ based fullerenes; (**b**) C_70_ based fullerenes. All spectra are normalized based on the intensity of their 0–1 transition peak at ~700 nm.

UV-Vis spectra of the blends and pure polymer, normalized based on the intensity of their 0-1 transition peak at ~700 nm, are depicted in Figure 5. The polymer PffBT4T-2OD is the main responsible for the light absorption and the fullerenes contribute only a minor part to the total absorbance of the active layer in the visible region. As shown in Figure 5, the pure polymer PffBT4T-2OD has essentially three main absorption bands in the visible region, with maxima located at ~460, 640 and 700 nm. The band at lower energy exhibits an absorption edge at ~750 nm, corresponding to an optical bandgap of ~1.65 eV [56], which is in perfect agreement with the literature [13]. Comparing Figure 5a,b it is evident that the BHJs using C_70_-based fullerenes display, in the 400–700 nm range, a stronger light absorption than the BHJs using C_60_-based fullerenes. This observation is expected considering that, as shown in Figure S25 in the Supplementary Information, the pure C_70_-based fullerenes display a stronger light absorption in the visible range. The higher light absorption of the C_70_-based BHJs should be a factor that, *per se*, obviously favours device efficiency assuming that all other factors remain the same. We discuss this question later.

**Figure 6:**
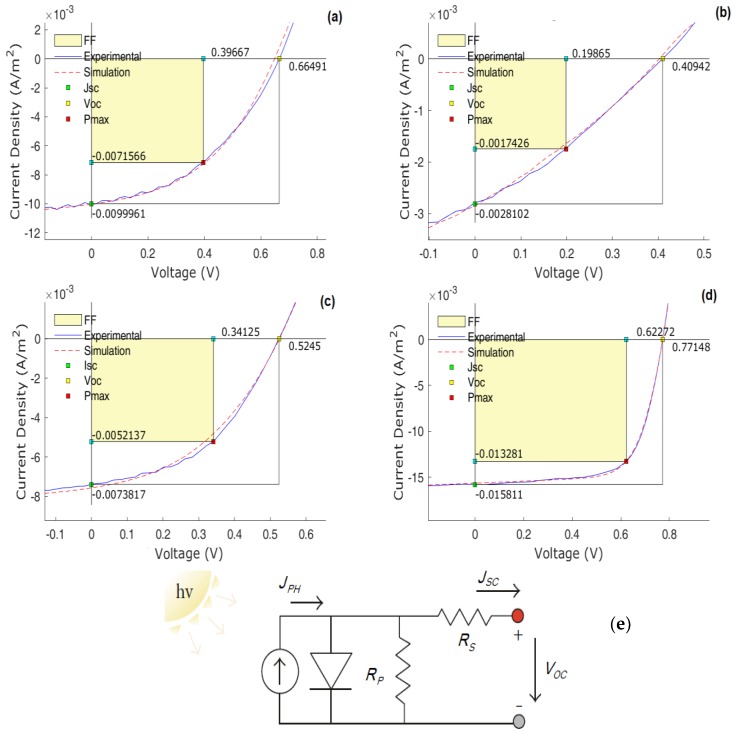
Full simulation data (dashed lines) for the electrical current density—applied voltage (J-V) for OPVs based in donors: (**a**) **60a**; (**b**) **60c**; (**c**) **70c**; (**d**) PC_71_BM. The general equivalent electric circuit used for simulation of the experimental data is represented in (**e**).

The current density-voltage (J–V) curves of devices were fitted to the usual OPV equivalent circuit employing genetic algorithms as previously described [57]. Figure 6 shows the most significant results. The full data is present in Table 4.

Several issues should be discussed regarding our macroscopic device physics data, as the influence of the space charge in the J–V data, mentioned above, reveals a departure from the ideal equivalent electrical circuit for the OPVs.

Concerning the values of *J_SC_*, it is clear that all of them are considerably lower than the value obtained with the standard PC_71_BM. In condition of *V* = 0, *J* should be given by $J\;=\;qdGP_{c}$ where *q* is the electronic charge, *d* the film thickness, *G* (depending on electrical current *I*) the photon flux by volume absorbed by the OPV and *P_C_* (depending on both electrical current *I* and applied voltage *V*) the conventional charge collection probability. The easiest way to estimate *P_C_* is by the ratio between the *J_SC_* and the reverse saturation current *J_PH_*. In our case, and due to the significant component of space charge J–V curve under high reverse bias, it is difficult to obtain experimentally the value of *J_PH_*. Therefore, the estimated values obtained from the equivalent circuit simulation with experimental data were used. As usual in OPVs, the values of *P_C_* are almost near 1 (considering the experimental error) but for the case of fullerene **60c**, a clear low value of *Pc* = 0.76 is obtained, corresponding also to the sample that provides the lower value of efficiency. With exception of the sample **70a** (*P_C_* = 0.89, η = 0.82), all the remaining values of the charge collection probability are higher than 0.92, with some absence of correlation with efficiency for some samples.

Regarding the *V_OC_* values for devices with the new fullerenes, all these are also lower than the value obtained with the standard PC_71_BM. A well-known [58] simple empirical relationship between *V_OC_* and the HOMO/LUMO levels of respectively donor/acceptor can be established, leading to $V_{OC}\approx \left(E_{LUMO}^{A}-E_{HOMO}^{D}\right)-0.3$. In practice, the search for a suitable explanation for the empirical 0.3 (V) in *V_OC_* equation, has shown that open-circuit voltage is dependent on the quasi-Fermi levels in both donor and acceptor materials. Cowan et al. [59] proposed a model that takes into account such quasi-Fermi levels influence, that can be described by

(1) $$V_{OC}=\frac{1}{q}\left(E_{LUMO}^{A}-E_{HOMO}^{D}-\Delta \right)-\frac{kT}{q}\ln\left(\frac{n_{e}n_{h}}{N_{c}^{2}}\right),$$

where *n_e_* and *n_h_* are respectively, the electron and hole densities in the fullerene and polymer domains at open circuit, and *N_c_* is the density of conduction states of the polymer and fullerene (assumed here to be equal) and Δ is an energy shift that originates from disorder within the phase-separated polymer and fullerene regions.

From the experimental data of our samples, and considering the HOMO/LUMO levels determined, we should expect significantly high values for *V_OC_*. However, the noticeable SCLC contribution in the J-V data, predicts a significant disorder in phase separation, as the simulation data indicates (a clear departing, in some samples, from the theoretical simple equivalent electrical circuit) due to the poor fit to the perfect diode model. This means that, in spite of interesting expectations for figures of merit-based in the HOMO/LUMO levels of some materials, the degradation of the active layer with further phase disorder, should be the suitable explanation for the low *V_OC_* values, as the energetic disorder contribution Δ must be significantly high. Additionally, the departing of the pure diode model (for phase separation) toward a device that becomes electrically governed by a space charge contribution gives rise to several other implications. In fact, the space charge influence in organic devices suggests an existence of intrinsic defects acting as energy traps for electrical carriers (see, for instance in our data, the clear lower values of *R_P_* obtained in all devices with new fullerenes compared to the PC_71_BM), that should imply a decrease of *J_SC_* and a lowering of the fill-factor. Interfacial trap energy levels at D:A phase is one of the most critical contributions for the OPV figures of merit. In practice, a high density of such trap states should reduce the *J_SC_* with a further reduction of fill-factor *FF*, additionally to the more closed J-V curve and giving experimental results sometimes far from the expected. Additionally, the presence of traps arising from intrinsic defects has another main consequence that is the decrease of electrical carrier mobility, as the trap-free Poole-Frenkel mobility becomes lower, affected by the final trap’s density. This should further decrease *J_SC_* and contribute negatively to efficiency. Another discussion related with *J_SC_* can be made, focused in a comparison of the blends absorption spectra and the values of short-circuit current. In fact, several of the new fullerenes exhibit superior light absorption in the blue-green spectral region when compared with traditional PCBM, but the *J_SC_* are consistently lower. This apparent contradiction can be explained, in a first approximation, by the substantially low charge separation efficiency (the *J_SC_* depends primarily on the product of absorption, charge separation and charge collection efficiencies) mainly due to the observed energetic disorder in our active layers. These effects include a high series resistance, low parallel resistance and low *FF*, as expected from a strong SCLC contribution to the J-V data, in line with our results.

**Figure 7:**
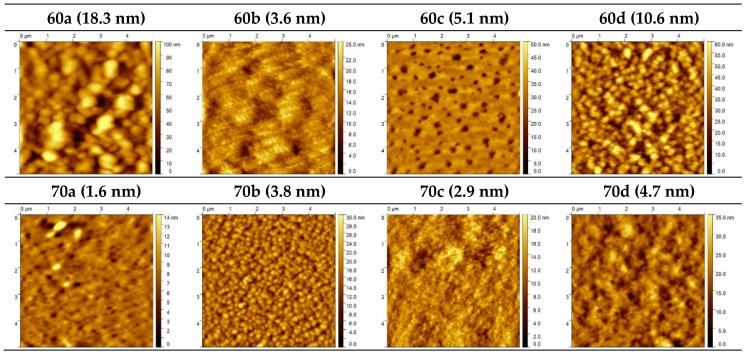
Atomic Force Microscopy (AFM) morphology images of PffBT4T-2OD based bulk-heterojunction films with the different fullerenes. The corresponding root mean square (*rms*) roughness values are indicated inside brackets.

Aiming to shed some light on the relationship between the nanoscopic BHJ morphology and the macroscopic device physics performance, the surface morphology of the PffBT4T-2OD:fullerene films was assessed using atomic force microscopy (AFM). The AFM height images are shown in Figure 7 and the corresponding phase images are shown in Figure S26. Also shown in Figure 7, inside brackets, are the corresponding values of root mean square (*rms*) roughness. However, the interpretation of these AFM images is very ambiguous and should be made with great caution. For example, if in one hand we can argue that BHJs with **60b** and **70c** have relatively higher efficiencies because they are among the smoother films with *rms* roughness of respectively 3.6 and 2.9 nm (supposedly with smaller fullerenes agglomerates); on the other hand, and in light of the same argument, it is hard to explain the higher efficiency of film **60a** which is rougher (rms roughness of 18.3 nm). We note however that AFM only probes the surface morphology of the film that may be very different from that of the underlying bulk material. Furthermore, as we explain in more detail below, the interpretation of an apparently coarse- or fine-grained physical microstructure alone can lead to incorrect conclusions regarding its effect on device performance. In fact, BHJs are morphologically complex systems and, beyond the measurement of the size of phase domains, their detailed characterization would also require the measurement of the degree of purity of the phase domains and the degree of orientation (crystallinity) inside those phase domains. However, such a detailed morphological characterization would require the use of some hardly accessible techniques such as Resonant Soft X-Ray Scattering (RSoXS) [60], Small Angle Neutron Scattering (SANS) [14,61], Grazing-Incidence Wide-Angle X-ray Scattering (GIWAXS) [62] and Neutron Reflectivity [63] and this is beyond the scope of our present work.

At this point, we note that fullerene functionalization can affect the efficiency of OPVs in different ways. Besides changing the HOMO/LUMO levels, different functional groups appended to the fulleropyrrolidines also affect the polymer-fullerene interactions and consequently the degree of fullerene dispersion in the polymer [64,65,66]. As a result, fullerenes with similar μ_e_ values in their pure state may originate BHJs with very different μ_e_ values due to different nanoscale morphologies [67,68]. The degree of fullerene dispersion in the polymer can also affect its LUMO level. For example, according to Durrant et al. [69], pure solid PC_61_BM has a 100–200 meV higher electron affinity than PC_61_BM finely dispersed in polystyrene, showing that fullerene aggregation consistently lowers its LUMO value and variations in the film microstructure can modulate the device V_OC_ by up ∼0.2 V [70]. Similarly, Lange et al. [71] suggested that the intimate contact between fullerenes and polymer chains in highly intermixed blends may reduce the LUMO level of fullerenes and cause a reduced *V_OC_*. Piersimoni et al. [72] reported that the crystallization of fullerene molecules lowers the energy of the charge transfer state (*E*_CT_) and this is directly manifested in a reduced *V_OC_*. Several studies have also found that a large degree of the energetic disorder can cause significant reductions in the achievable *V_OC_* in OPVs [73,74], as we have already discussed. Troshin et al. [75] observed that the fullerene solubility strongly affects the BHJ morphology and consequently the solar cell figures of merit including the *V_OC_*. This can be attributed to the density of states (DOS) near the HOMO/LUMO levels at D:A interface, changed by the bulk phase separation morphology (and consequently with intrinsic defects formation) and modifying the quasi-Fermi levels with further change in *V_OC_*. *V_OC_* was shown to attain maximum values for fullerene solubilities ≥ 30 mg·mL^−1^. Different functional groups attached to the C_60_ and C_70_ cages, can also severely change the electronic coupling between adjacent fullerenes through steric and/or electronic effects as well as their solid-state packing and crystallization properties, causing a significant decrease in their local electron mobility [76]. Additionally, the structural and energetic disorder that results from the presence of different fullerene isomers, as we have found in our C_70_-based fullerenes, was also shown to contribute to the low electron mobility often observed in the fullerene-rich phase [50,77,78] with the consequent detrimental effect in the performance of devices. As such, efforts are underway to devise regioselective synthetic pathways [43,44,79,80,81].

Finally, we mention that a relatively recent study from 2017, by Karakawa et al., suggests that fulleropyrrolidines, due to their high electron density on the *N*-atom of pyrrolidine, are intrinsically basic and can have a detrimental interaction with the acidic PEDOT:PSS layer, that results in the degradation of the photovoltaic performance [82]. Based on this rationale, lowering the electron density on the *N*-atom of pyrrolidine, i.e., lowering its basicity, can be effective at improving the device performance. This study by Karakawa et al. also referred us to some other previous studies reporting similar results but, according to these authors, not adequately explained [54,83,84]. In 2014, the same authors [83] had tested *N*-alkyl- and *N*-phenyl-fulleropyrrolidines in P3HT based OPV devices with the standard architecture ITO/PEDOT:PSS/BHJ/Al. OPV cells based on the *N*-alkyl-fulleropyrrolidines (more basic) exhibited low performances, while the performances obtained using the *N*-phenyl-fulleropyrrolidines (less basic) were comparable to or even higher than that of the corresponding PC_61_BM-based devices. Also in 2014, Pitliya et al. [84] tested a novel *N*-alkyl-fulleropyrrolidine, containing a -CN group, in P3HT based devices with the standard architecture ITO/PEDOT:PSS/BHJ/Al an obtained efficiencies (1.77%) only slightly lower than reference PC_61_BM-based devices (2.14%). In the light of the work of Karakawa et al. the relatively good efficiencies obtained by Pitliya et al. can be explained by the electron withdrawing -CN group that reduces the basicity of the pyrrolidine. However, and as far as we know, this is the only study in the literature in which devices with standard architecture ITO/PEDOT:PSS/BHJ/Al and based on *N*-alkyl-fulleropyrrolidine acceptors achieved efficiencies comparable to reference PC_61_BM-based devices. In 2015 Liang et al. [54] also reported that *N*-phenyl-fulleropyrrolidines produced higher-performing devices than *N*-alkyl-fulleropyrrolidines, in accordance with the work of Karakawa et al. [83].

In our present work, the phenyl substituents -COOCH_3_ and -F are electron-withdrawing groups that decrease the electron density on the *N*-atom of pyrrolidine (less basic) and the -OCH_3_ substituent is an electron donor group that increases the electron density on the *N*-atom of pyrrolidine (more basic). Therefore, the basicity of the different C_60_-fulleropyrrolidines should increase in the order, **60c** ~ **60d** < **60a** < **60b**, and a similar order should be observed for the corresponding C_70_-fulleropyrrolidines. However, in our opinion, the effect of these functional groups on the basicity of the pyrrolidine should be small because they are not directly bonded to the *N* atom. In fact, when we compare these fullerene basicity rankings with our device data, we conclude that there is no obvious correlation between them. For example, in the case of C_60_-fulleropyrrolidines we observe that the most basic acceptors **60a** and **60b**, originate the higher efficiency devices which apparently goes against Karakawa et al. [82]. In the present work, we have obtained low efficiencies for all the fulleropyrrolidine-based devices, very likely due to the detrimental PEDOT-PSS/*N*-methyl-fulleropyrrolidine interfacial interaction, and we believe that much better performances could be obtained if we had tested devices with inverted architecture (i.e., without PEDOT:PSS). However, the relative efficiency of our devices does not conform to the relative basicity of the corresponding fulleropyrrolidines which shows that other factors, as discussed above, must also play their important role. This very likely detrimental effect of the PEDOT:PSS layer on our *N*-methyl-fulleropyrrolidine based devices is a very interesting problem that we will carefully address in a future study.

## 4. Conclusions

Novel C_60_ and C_70_ fullerene derivatives were synthesized by the well-known Prato reaction and the corresponding HOMO/LUMO levels measured by voltammetry. Despite the favourable LUMO levels of some of these novel fullerenes, compared to PC_71_BM, all of them originated PffBT4T-2OD-based devices with poorer performances than reference devices based on the standard PC_71_BM. The functionalization of C_70_ yielded a mixture of four regioisomers (α1, α2, β1, β2) that have different HOMO/LUMO levels as determined by DFT calculations. Interestingly, these C_70_ derivatives despite their higher light absorption in the visible range originated devices with lower performances than the corresponding C_60_ derivatives. This lower performance of devices based on C_70_ derivatives is most likely a consequence of both the energetic disorder introduced by the mixture of regioisomers and the presence in a relatively high amount of the β1-isomer which, according to recent reports, is known to be particularly harmful for device performance. In fact, energetic disorder, especially at D:A interface, tends to degrade the *V_OC_*, and (also in the bulk) introduces intrinsic defects responsible for low *J_SC_* (including a high influence of SCLC trap-dependent behaviour with also further decrease of carrier mobility) and increasing the exciton loss by recombination. While is not possible to make any straightforward correlation between the quantity of β1-isomer and the values of efficiency observed for each C_70_ derivative, it is possible to note that the amounts of β1 are high enough to interact negatively in the film blends decreasing their overall efficiency when compared with C_60_ derivatives.

## Figures and Tables

**Table 1:** Fraction in percentage of the isomers present in derivatives **70a**–**70d.**

	α1 (%)	α2 (%)	β1 (%)	β2 (%)	α:β (%)
**70a**	30	17	27	26	47:53
**70b**	28	21	29	22	49:51
**70c**	36	19	26	19	55:45
**70d**	39	18	24	18	57:43

**Table 2:** Experimental and computed HOMO and LUMO energies at the PBE-def2-TZVP/PBEh-3c level. All values in eV.

	HOMO			LUMO		
	Experimental	Computed		Experimental	Computed	
**PC_61_BM**	−5.97	−5.47		−3.89	−3.94	
**60a**	−5.74	−5.42		−3.87	−3.90	
**60b**	−5.72	−5.38		−3.86	−3.87	
**60c**	−5.54	−5.46		−3.87	−3.94	
**60d**	−5.83	−5.53		−3.97	−4.00	
		α	β		α	β
**PC_71_BM**	−5.87	−5.52	−5.44	−3.89	−3.85	−3.87
**70a**	−5.68	−5.47	−5.40	−3.99	−3.80	−3.85
**70b**	−5.64	−5.38	−5.34	−4.05	−3.76	−3.81
**70c**	−5.51	−5.52	−5.45	−3.87	−3.85	−3.90
**70d**	−5.77	−5.56	−5.48	−3.91	−3.88	−3.92

**Table 3:** Device metrics showing the peak and (average) values for PCE for devices prepared using different fullerene derivatives.

	PCE (%)	V_OC_ (V)	FF (%)	J_sc_ (mA/cm^2^)
**PC_71_BM**	8.41 (8.19 ± 0.24)	0.77 (0.74 ± 0.02)	71.2 (69.8 ± 1.7)	16.42 (15.87 ± 0.40)
**60a**	2.84 (2.54 ± 0.26)	0.66 (0.62 ± 0.04)	42.9 (49.2 ± 6.7)	9.96 (8.47 ± 1.51)
**60b**	2.04 (1.80 ± 0.17)	0.54 (0.54 ± 0.02)	38.8 (38.4 ± 2.8)	9.65 (8.65 ± 1.00)
**60c**	0.36 (0.33 ± 0.02)	0.56 (0.45 ± 0.07)	20.5 (27.7 ± 5.9)	2.80 (2.74 ± 0.38)
**60d**	0.98 (0.83 ± 0.10)	0.50 (0.50 ± 0.04)	44.8 (43.3 ± 4.5)	4.41 (3.98 ± 0.82)
**70a**	0.82 (0.73 ± 0.08)	0.54 (0.64 ± 0.22)	28.6 (28.6 ± 9.6)	5.30 (4.32 ± 0.67)
**70b**	1.12 (1.11 ± 0.01)	0.56 (0.54 ± 0.01)	27.2 (26.9 ± 0.4)	7.33 (7.34 ± 0.02)
**70c**	1.76 (1.72 ± 0.04)	0.52 (0.51 ± 0.01)	45.9 (44.6 ± 3.1)	7.36 (7.50 ± 0.44)
**70d**	0.77 (0.70 ± 0.06)	0.54 (0.52 ± 0.01)	41.1 (41.1 ± 0.6)	3.48 (3.24 ± 0.23)

**Table 4:** Generated photocurrent (*J_ph_*), parallel (*R_p_*), and series resistance (*R_s_*) obtained by the equivalent circuit fit to the experimental data.

	*J_ph_* (mA/cm^2^)	*R_s_* (Ω)	*R_p_* (Ω)
**PC_71_BM**	15.66	159	9.72 × 10^4^
**60a**	10.20	579	2.00 × 10^4^
**60b**	9.64	475	1.69 × 10^4^
**60c**	3.67	3724	1.00 × 10^4^
**60d**	4.76	866	1.49 × 10^4^
**70a**	5.98	1771	0.84 × 10^4^
**70b**	7.93	1028	0.56 × 10^4^
**70c**	7.69	587	2.04 × 10^4^
**70d**	3.67	1139	1.55 × 10^4^

## Supplementary Materials

The following are available online at https://www.mdpi.com/1996-1944/12/24/4100/s1, Figure S1: 1H NMR spectrum of compound **60a** in a mixture of CS2 and acetone-d6, Figure S2: ^13^C NMR spectrum of compound **60a** in a mixture of CS_2_ and acetone-d6, Figure S3: HSQC spectrum of compound **60a** in a mixture of CS_2_ and acetone-d6, Figure S4: ^1^H NMR spectrum of compound **60b** in a mixture of CS_2_ and acetone-d6, Figure S5: ^13^C NMR spectrum of compound **60b** in a mixture of CS_2_ and acetone-d6, Figure S6: HSQC spectrum of compound **60b** in a mixture of CS_2_ and acetone-d6, Figure S7: ^1^H NMR spectrum of compound **60c** in a mixture of CS_2_ and acetone-d6, Figure S8: ^13^C NMR spectrum of compound **60c** in a mixture of CS_2_ and acetone-d6, Figure S9: HSQC spectrum of compound **60c** in a mixture of CS_2_ and acetone-d6, Figure S10: ^1^H NMR spectrum of compound **70a** in a mixture of CS_2_ and acetone-d6, Figure S11: ^13^C NMR spectrum of compound **70a** in a mixture of CS_2_ and acetone-d6, Figure S12: HSQC spectrum of compound **70a** in a mixture of CS_2_ and acetone-d6, Figure S13: ^1^H NMR spectrum of compound **70b** in a mixture of CS_2_ and acetone-d6, Figure S14: ^13^C NMR spectrum of compound **70b** in a mixture of CS_2_ and acetone-d6, Figure S15: HSQC spectrum of compound **70b** in a mixture of CS_2_ and acetone-d6, Figure S16: ^1^H NMR spectrum of compound **70c** in a mixture of CS_2_ and acetone-d6, Figure S17: ^13^C NMR spectrum of compound **70c** in a mixture of CS_2_ and acetone-d6, Figure S18: HSQC spectrum of compound **70c** in a mixture of CS_2_ and acetone-d6, Figure S19: ^1^H NMR spectrum of compound **60d** in a mixture of CS_2_ and acetone-d6, Figure S20: ^13^C NMR spectrum of compound **60d** in a mixture of CS_2_ and acetone-d6, Figure S21: HSQC spectrum of compound **60d** in a mixture of CS_2_ and acetone-d6, Figure S22: ^1^H NMR spectrum of compound **70d** in a mixture of CS_2_ and acetone-d6, Figure S23: ^13^C NMR spectrum of compound **70d** in a mixture of CS_2_ and acetone-d6, Figure S24: HSQC spectrum of compound **70d** in a mixture of CS_2_ and acetone-d6, Figure S25: UV-Vis spectroscopy of the fullerenes in 1,2-dichlorobenzene, Figure S26: AFM phase images of PffBT4T-2OD based bulk-heterojunction films with the different fullerenes, Table S1: HOMO and LUMO levels for all materials as calculated from cyclic voltammetry. The potential onsets used for the calculations are also indicated.
