# Supplementary material for: PffBT4T-2OD Based Solar Cells with Aryl-Substituted N-Methyl-Fulleropyrrolidine Acceptors
Source: Materials (Basel). 2019 Dec 8;12(24):4100. doi: 10.3390/ma12244100 (PMC6947311; doi:10.3390/ma12244100)
Supplement: Supplementary file 1 [file materials-12-04100-s001.pdf]

materials

Supplementary Information

PffBT4T-2OD Based Solar Cells with Aryl-Substituted *N*-Methyl-Fulleropyrrolidine Acceptors

Table S1. HOMO and LUMO levels for all materials as calculated from cyclic voltammetry. The potential onsets used for the calculations are also indicated.

Material	E_{ox}^{onset} (V)	HOMO (eV)	E_{red}^{onset} (V)	LUMO (eV)
PffBT4T-2OD	-	−5.34	-	−3.69
PC61BM	1.07	−5.97	−1.01	−3.89
PC71BM	0.97	−5.87	−1.01	−3.89
60a	0.84	−5.74	−1.03	−3.87
60b	0.82	−5.72	−1.04	−3.86
60c	0.64	−5.54	−1.03	−3.87
60d	0.93	−5.83	−0.93	−3.97
70a	0.78	−5.68	−0.91	−3.99
70b	0.74	−5.64	−0.85	−4.05
70c	0.81	−5.71	−1.03	−3.87
70d	0.87	−5.77	−0.99	−3.91

1. NMR Characterization

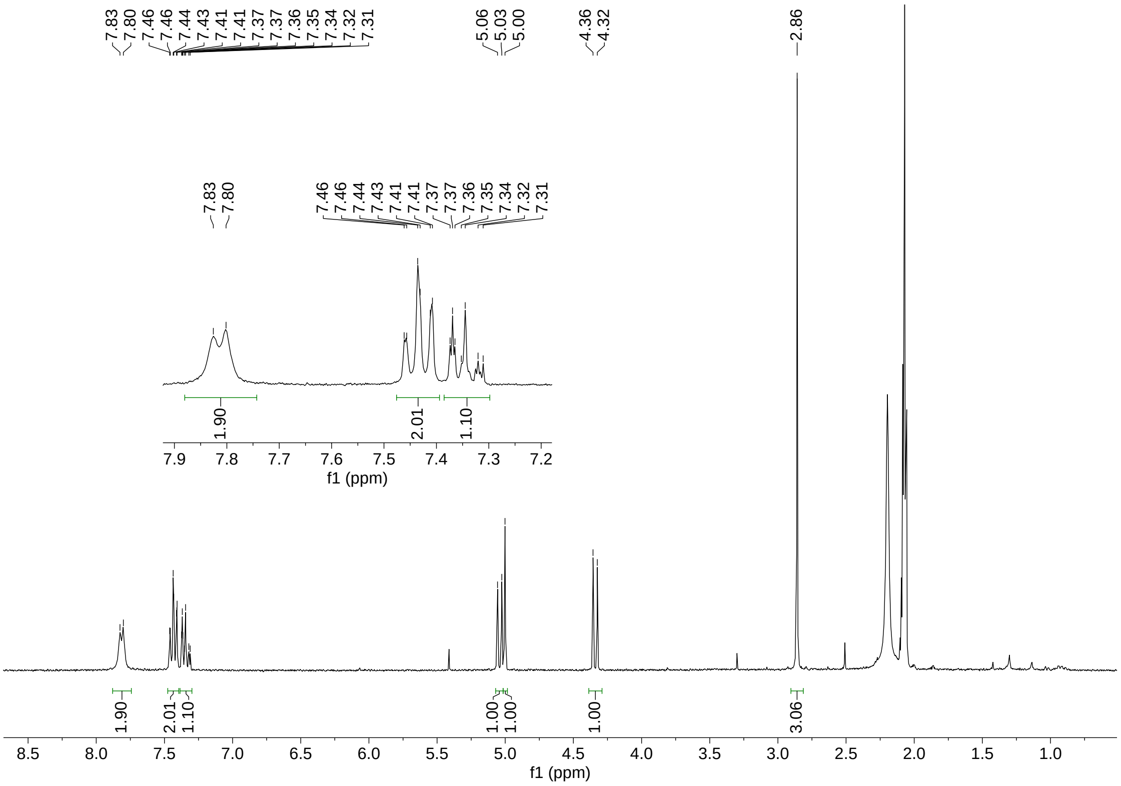

Figure S1. 1H NMR spectrum of compound **60a** in a mixture of CS2 and acetone-d6.

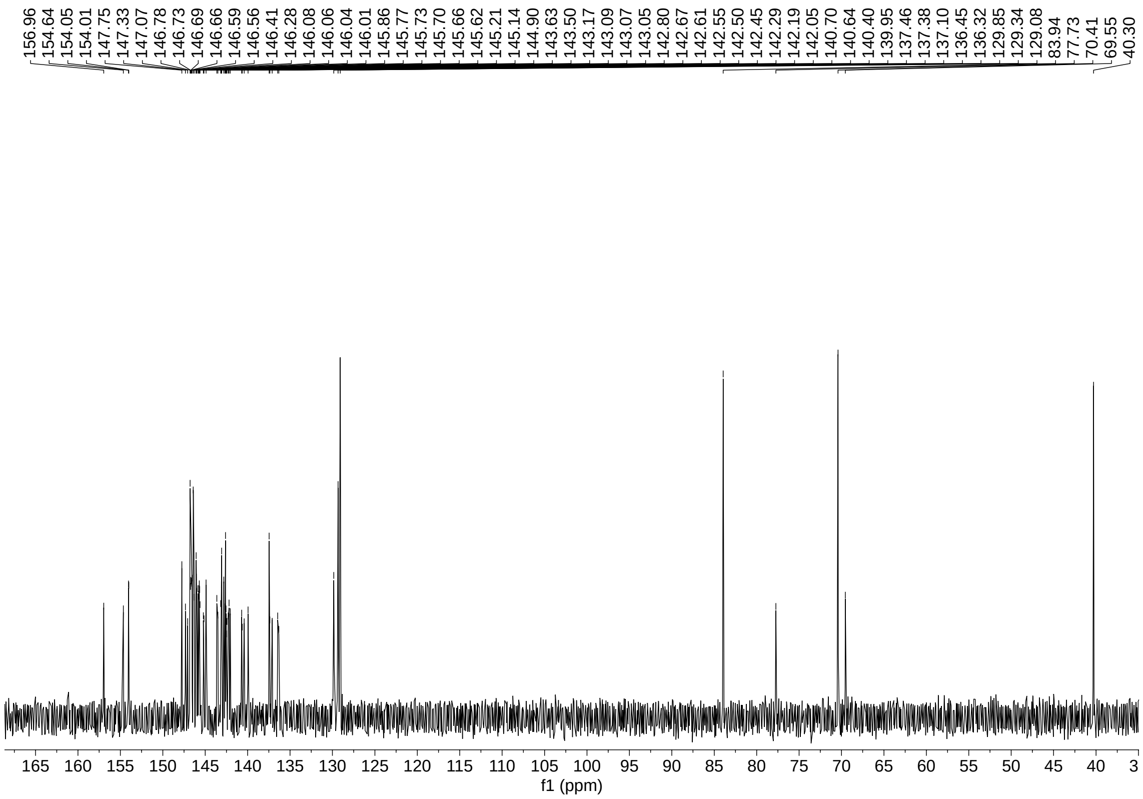

Figure S2. ^{13}C NMR spectrum of compound **60a** in a mixture of CS_2 and acetone-d6.

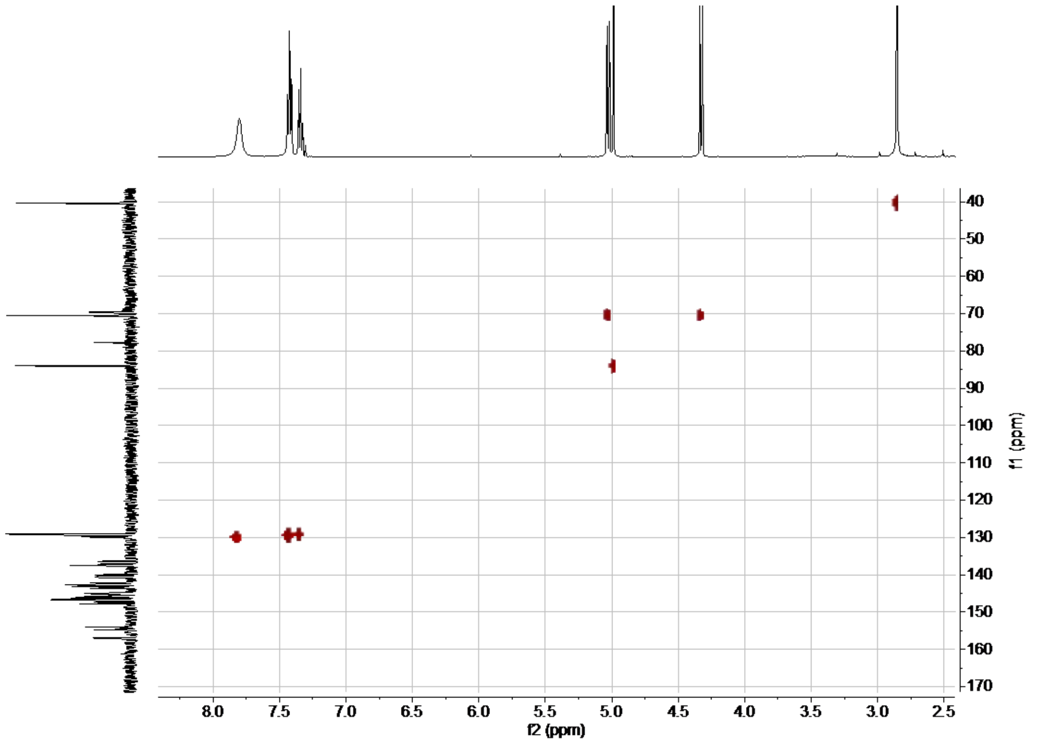

Figure S3. HSQC spectrum of compound **60a** in a mixture of CS_2 and acetone-d6.

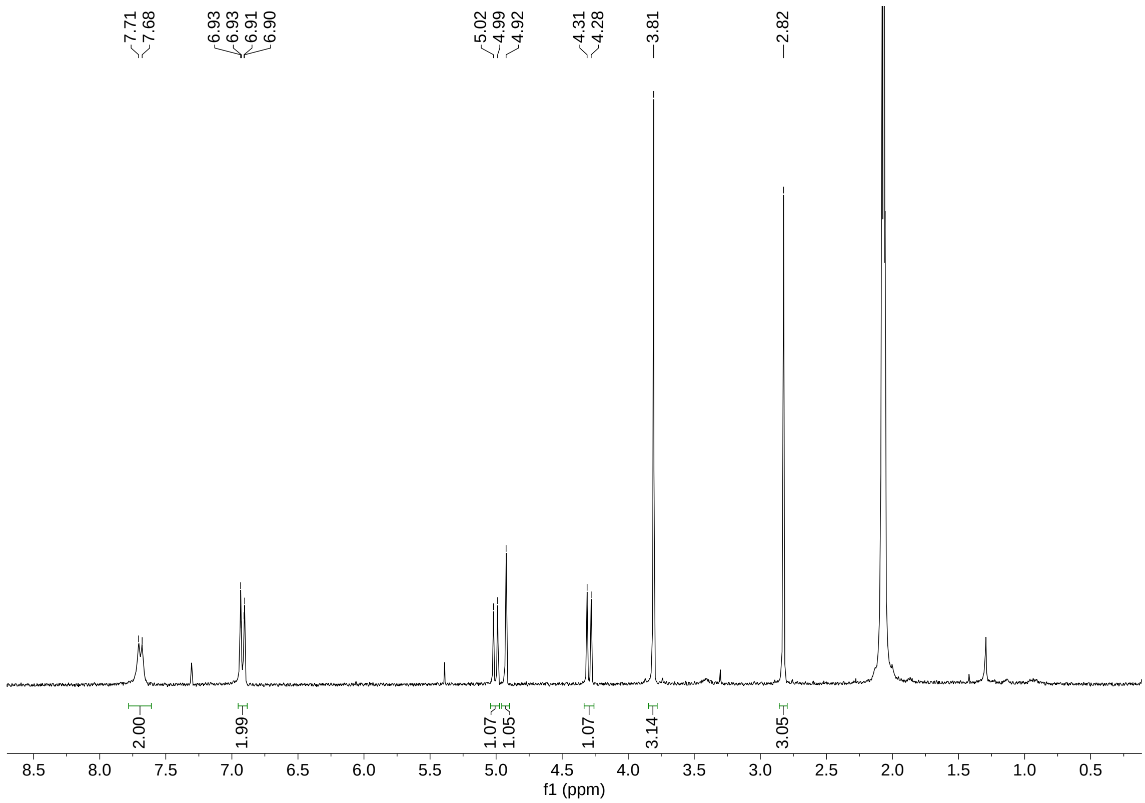

Figure S4. ${}^{1}H$ NMR spectrum of compound **60b** in a mixture of CS_2 and acetone-d6.

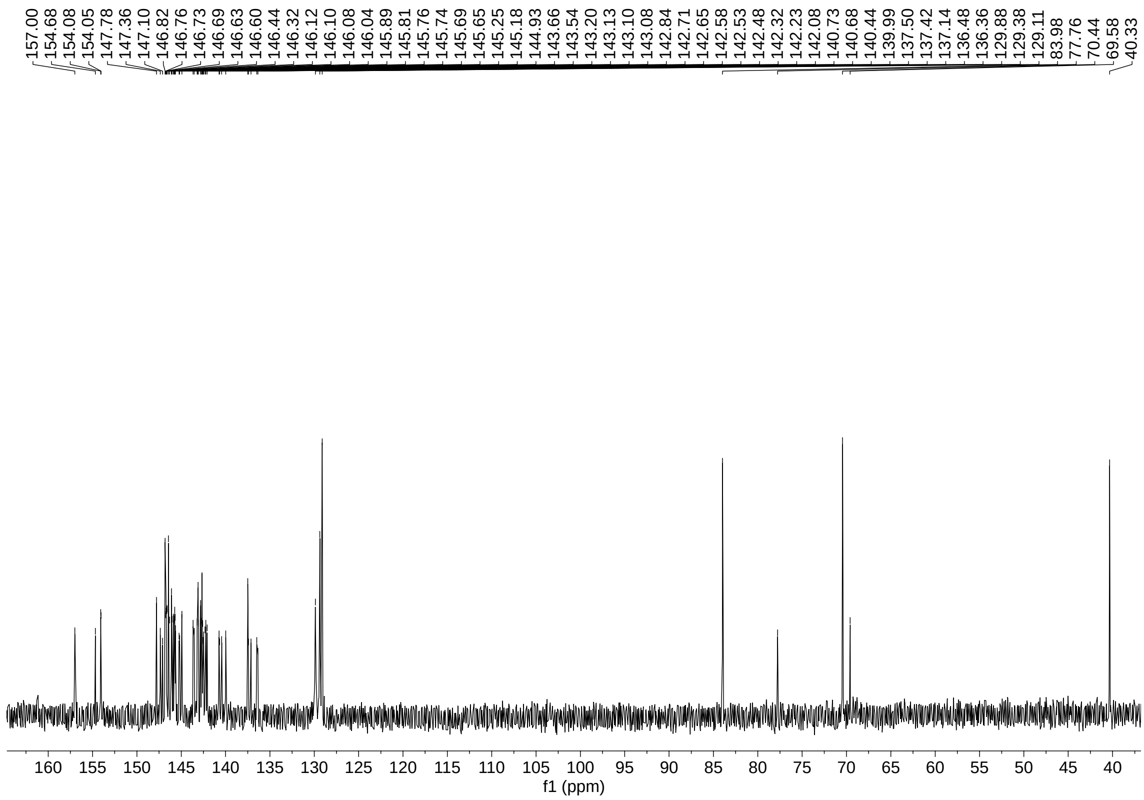

Figure S5. ${}^{13}C$ NMR spectrum of compound **60b** in a mixture of CS_2 and acetone-d6.

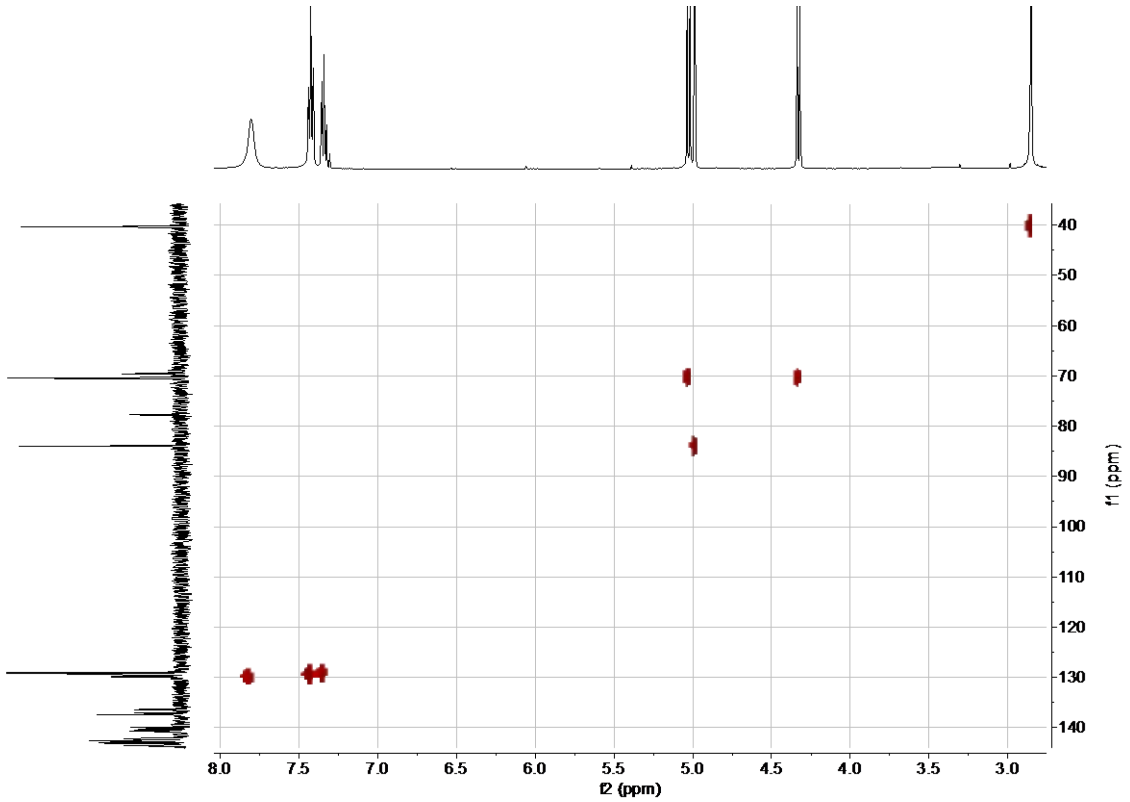

Figure S6. HSQC spectrum of compound **60b** in a mixture of CS_2 and acetone-d6.

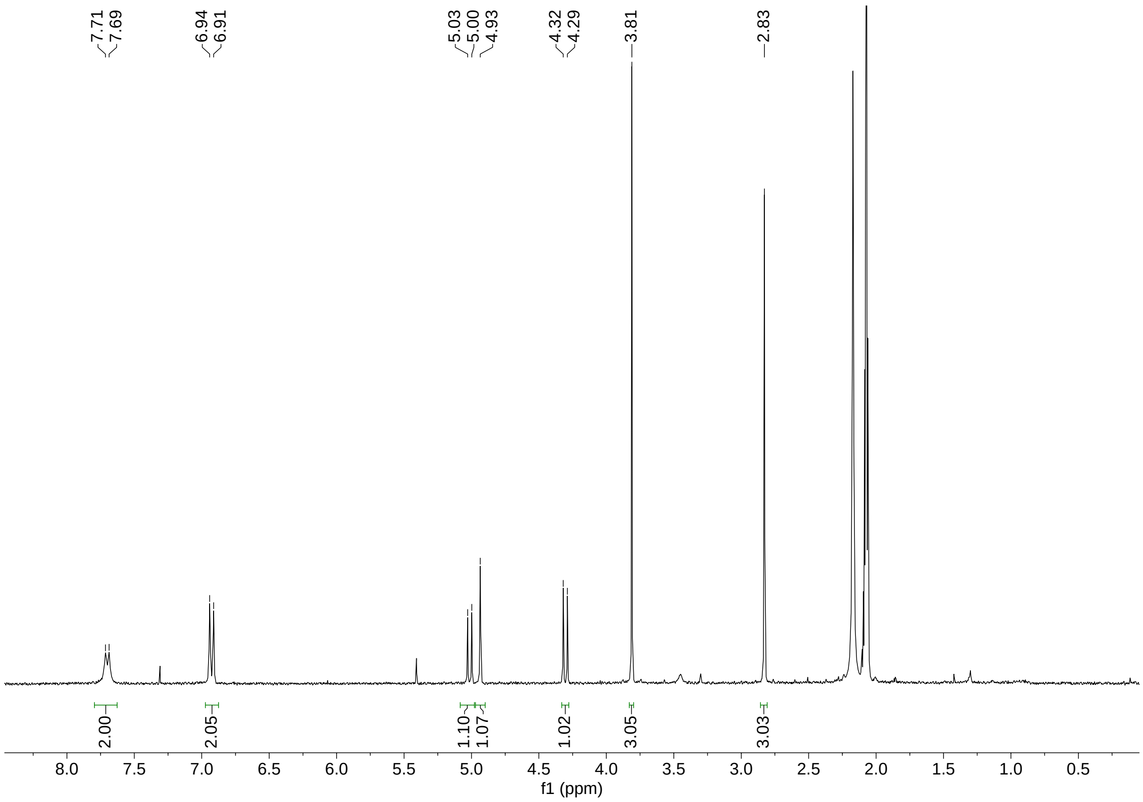

Figure S7. ^{1}H NMR spectrum of compound **60c** in a mixture of CS_2 and acetone-d6.

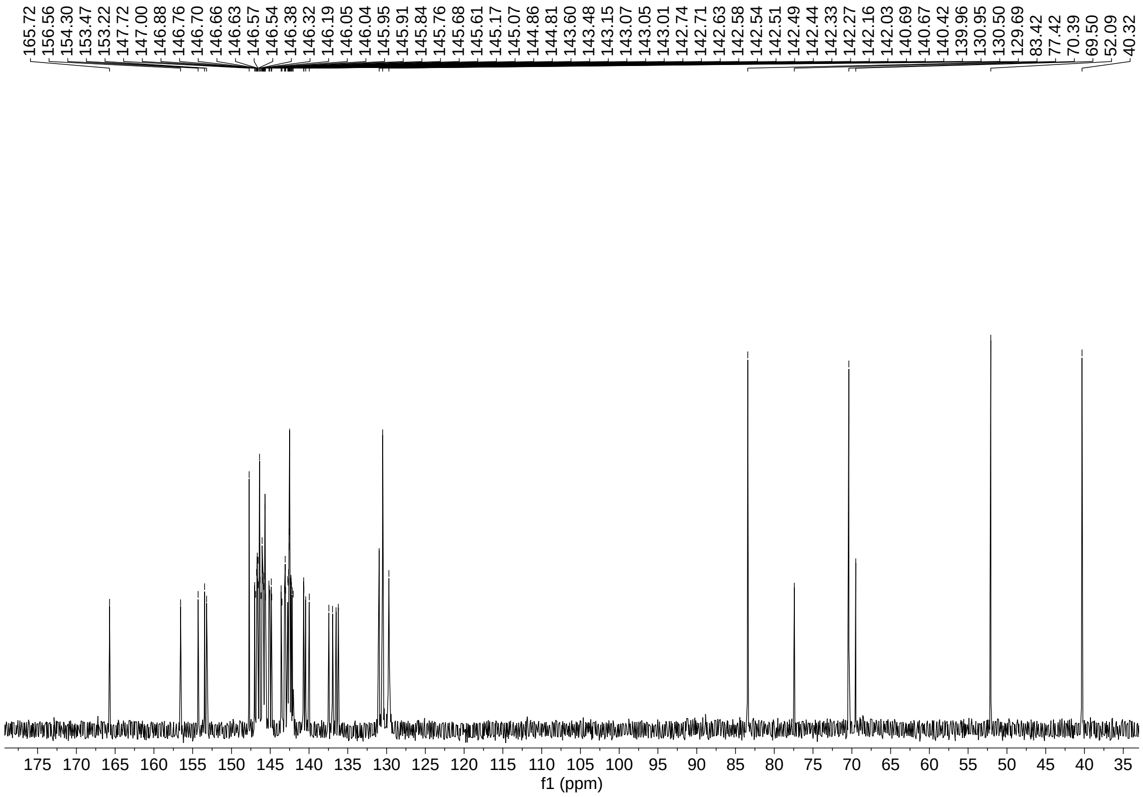

Figure S8. ^{13}C NMR spectrum of compound **60c** in a mixture of CS_2 and acetone-d6.

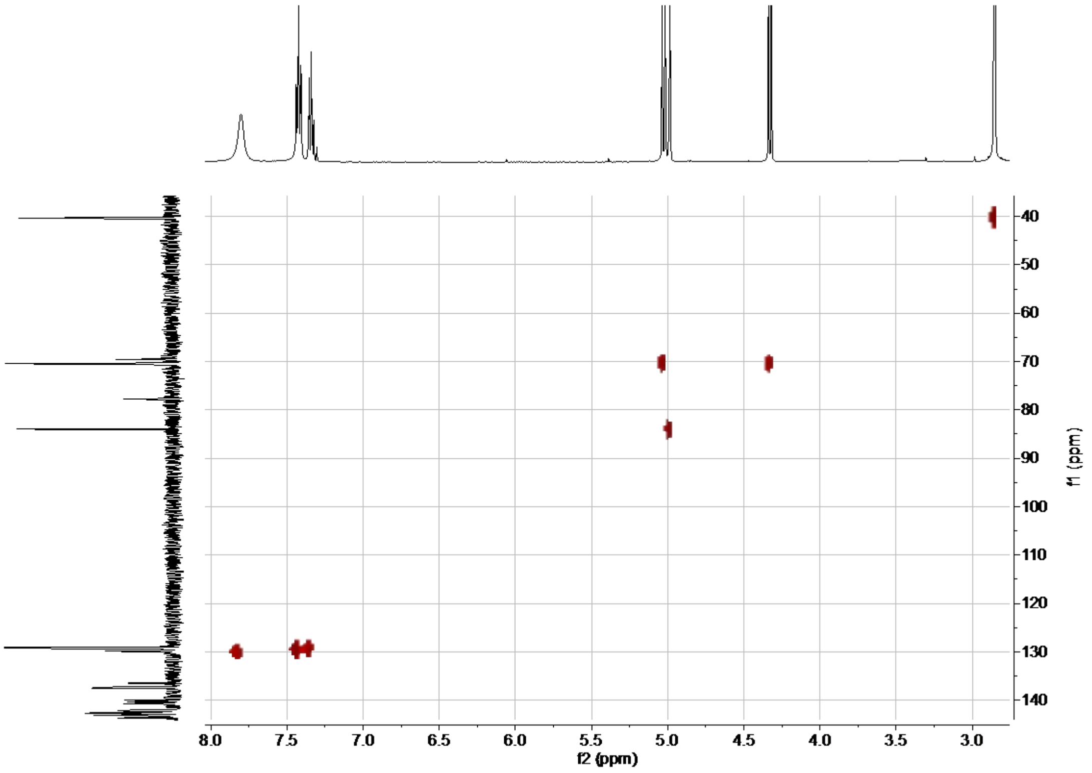

Figure S9. HSQC spectrum of compound **60c** in a mixture of CS_2 and acetone-d6.

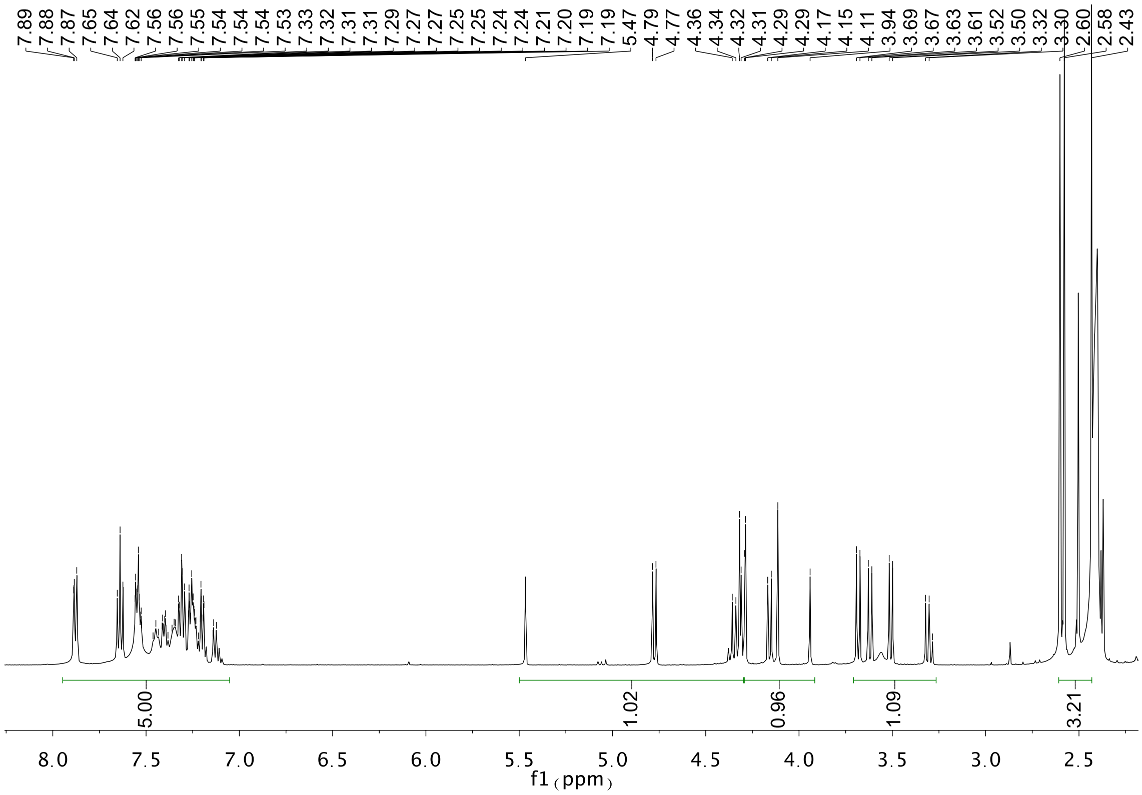

Figure S10. ^{1}H NMR spectrum of compound **70a** in a mixture of CS_2 and acetone-d6.

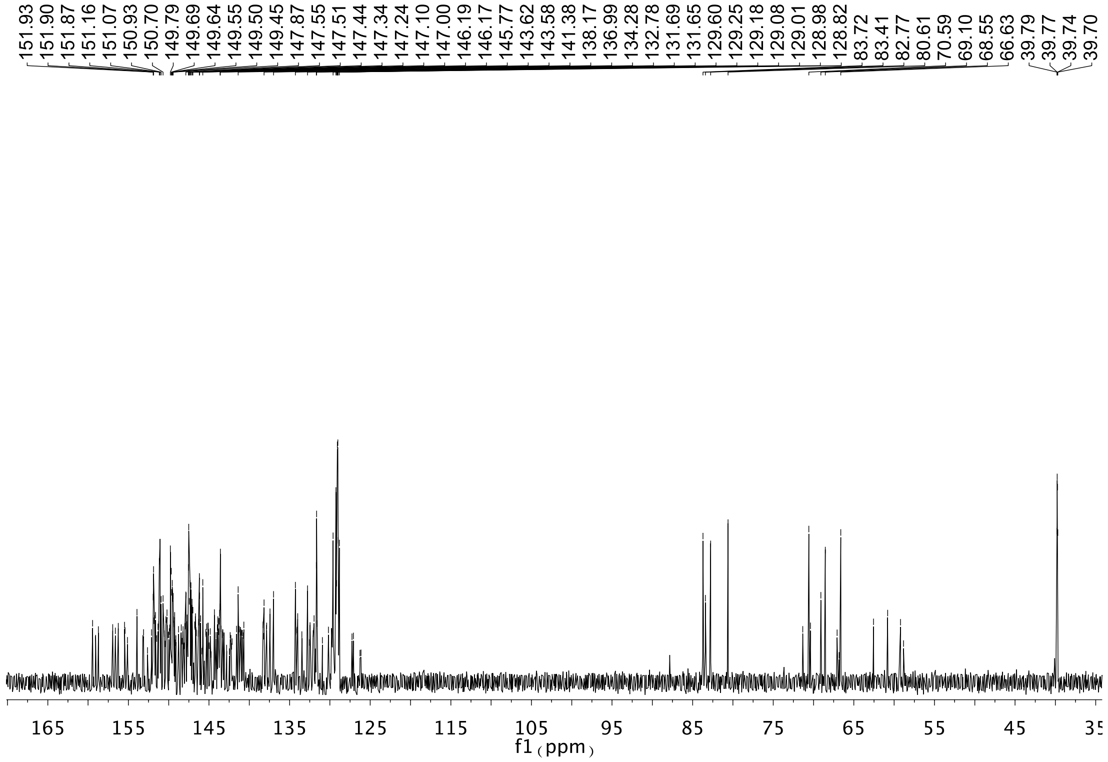

Figure S11. ^{13}C NMR spectrum of compound **70a** in a mixture of CS_2 and acetone-d6.

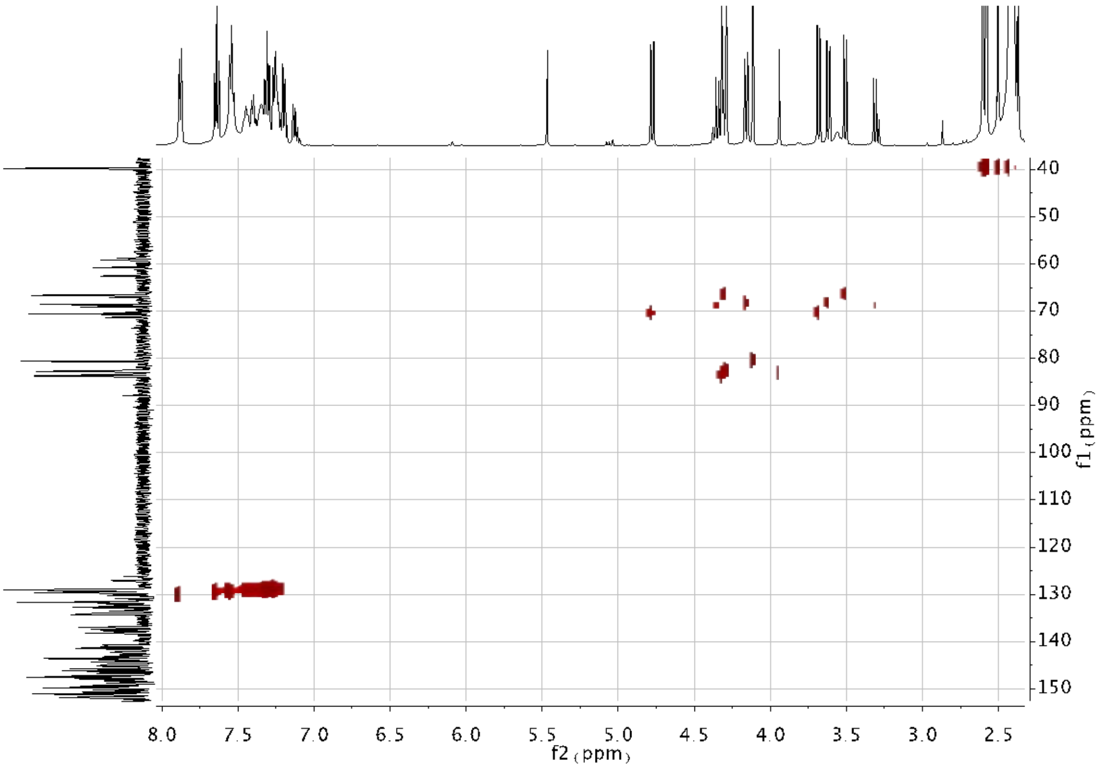

Figure S12. HSQC spectrum of compound **70a** in a mixture of CS_2 and acetone-d6.

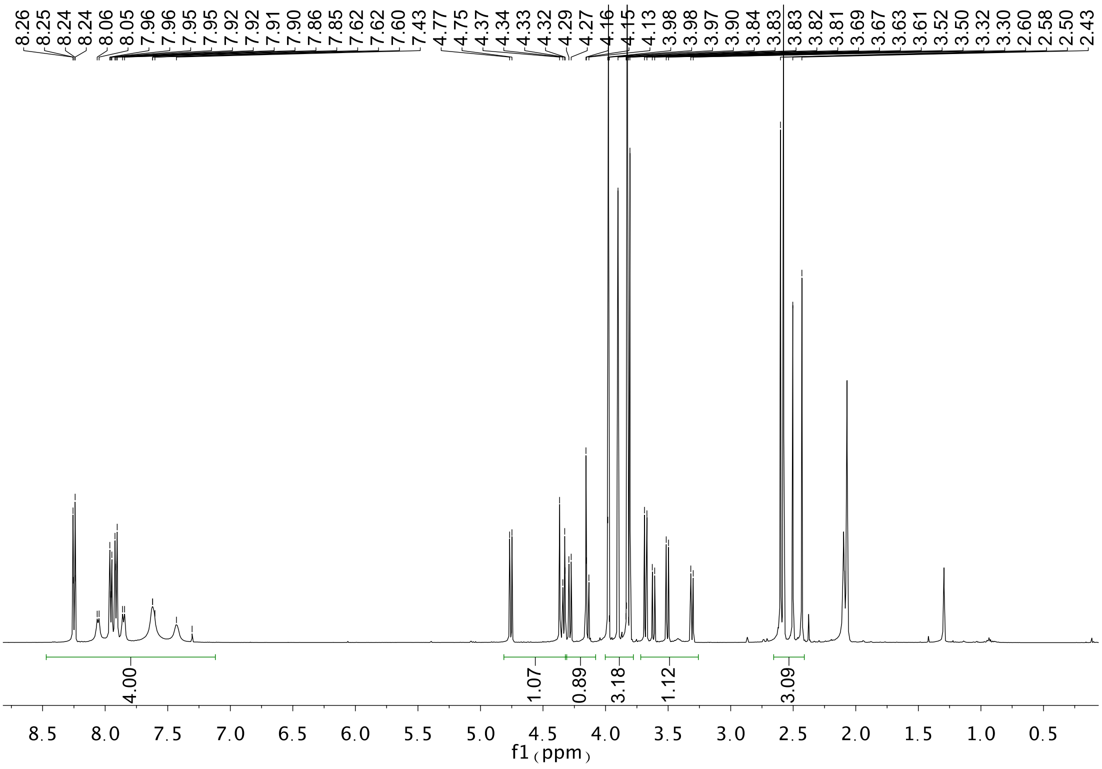

Figure S13. ^{1}H NMR spectrum of compound **70b** in a mixture of CS_2 and acetone-d6.

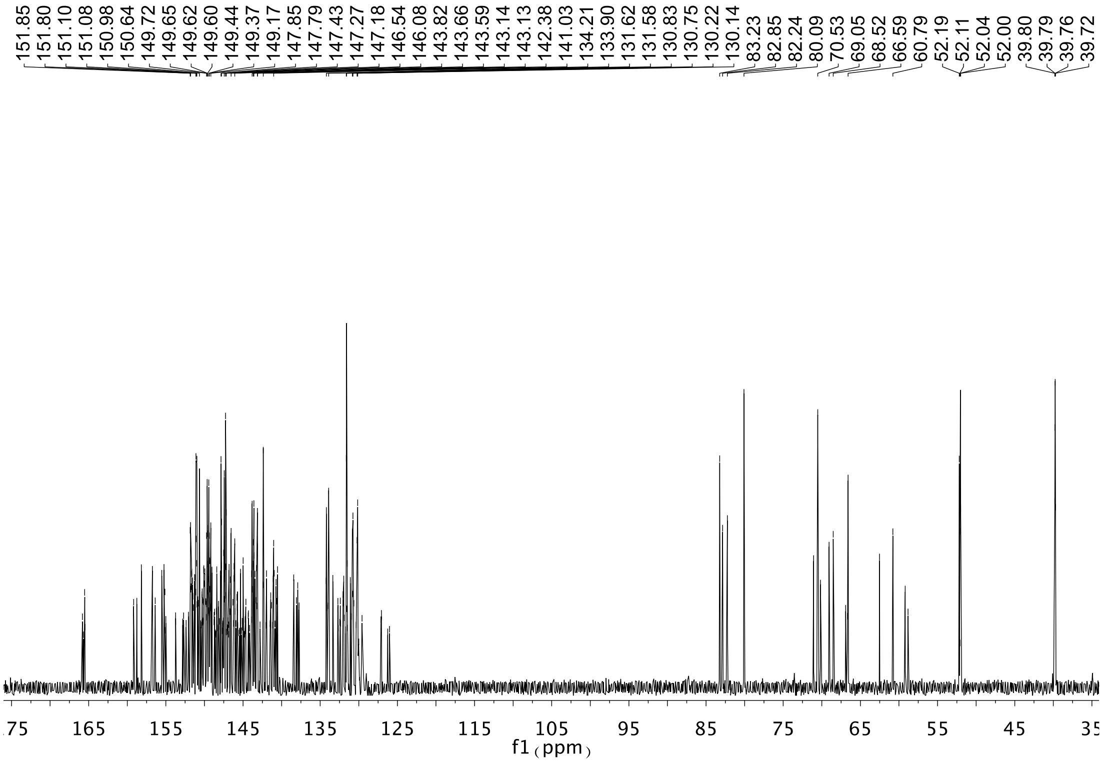

Figure S14. ^{13}C NMR spectrum of compound **70b** in a mixture of CS_2 and acetone-d6.

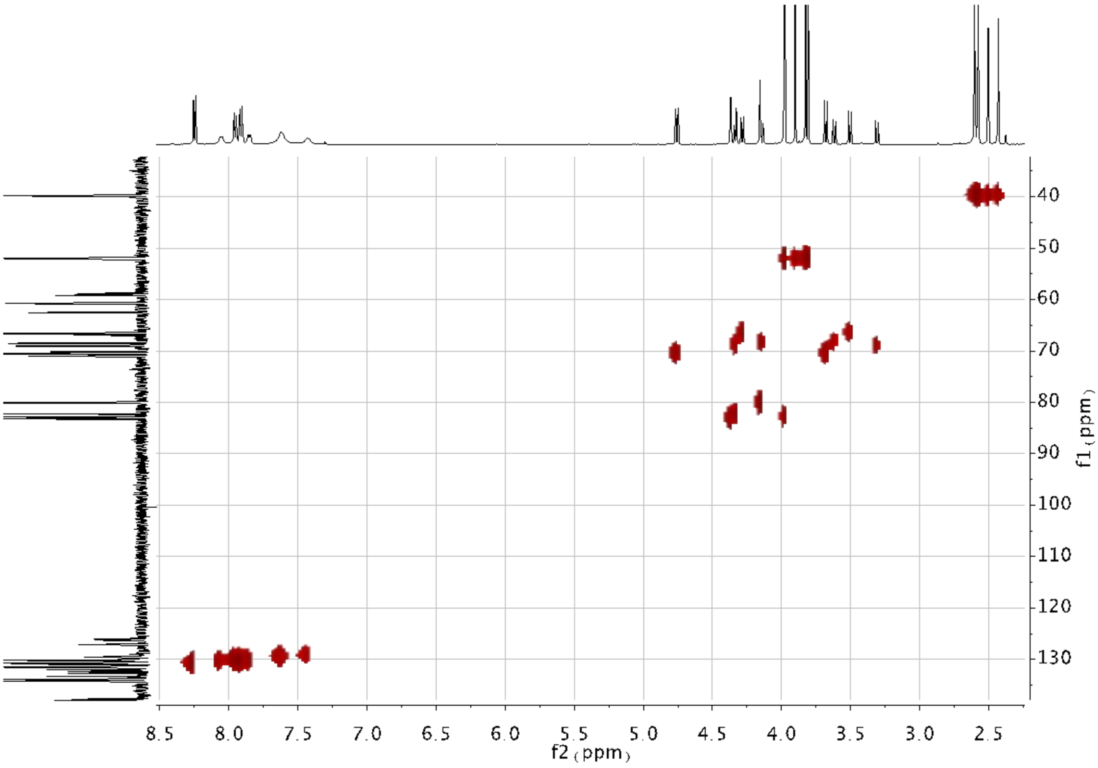

Figure S15. HSQC spectrum of compound **70b** in a mixture of CS_2 and acetone-d6.

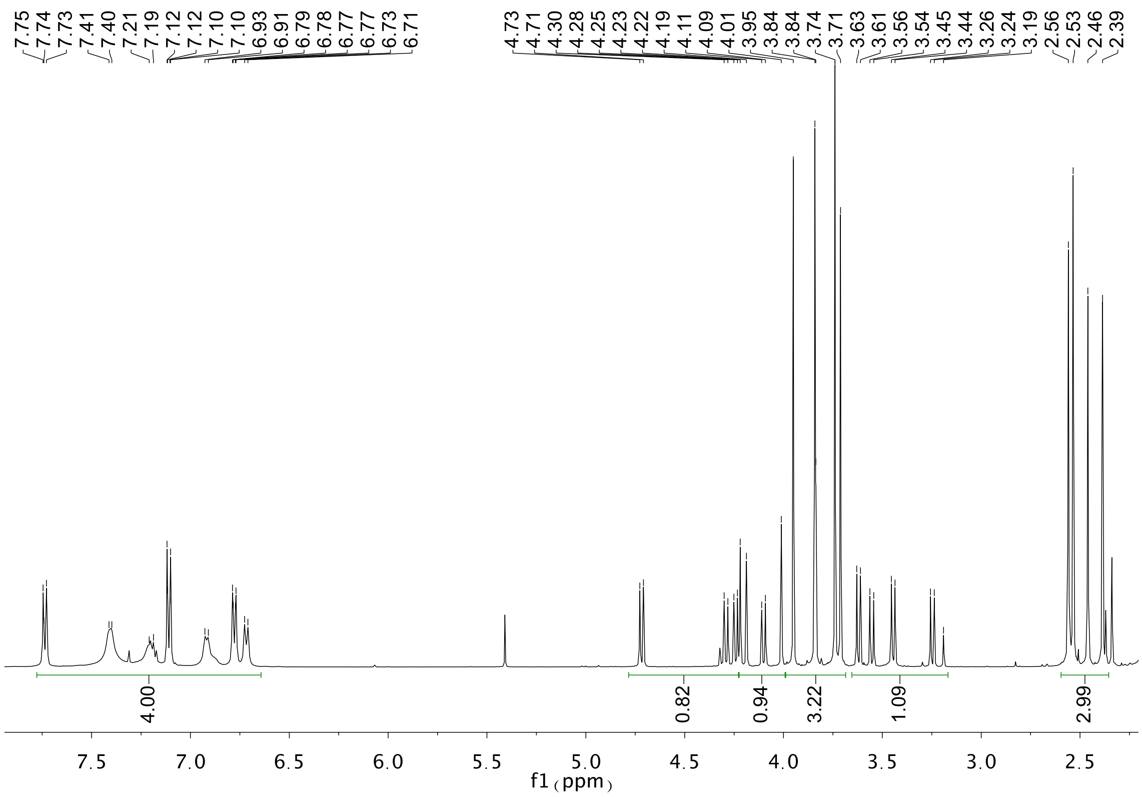

Figure S16. ^{1}H NMR spectrum of compound **70c** in a mixture of CS_2 and acetone-d6.

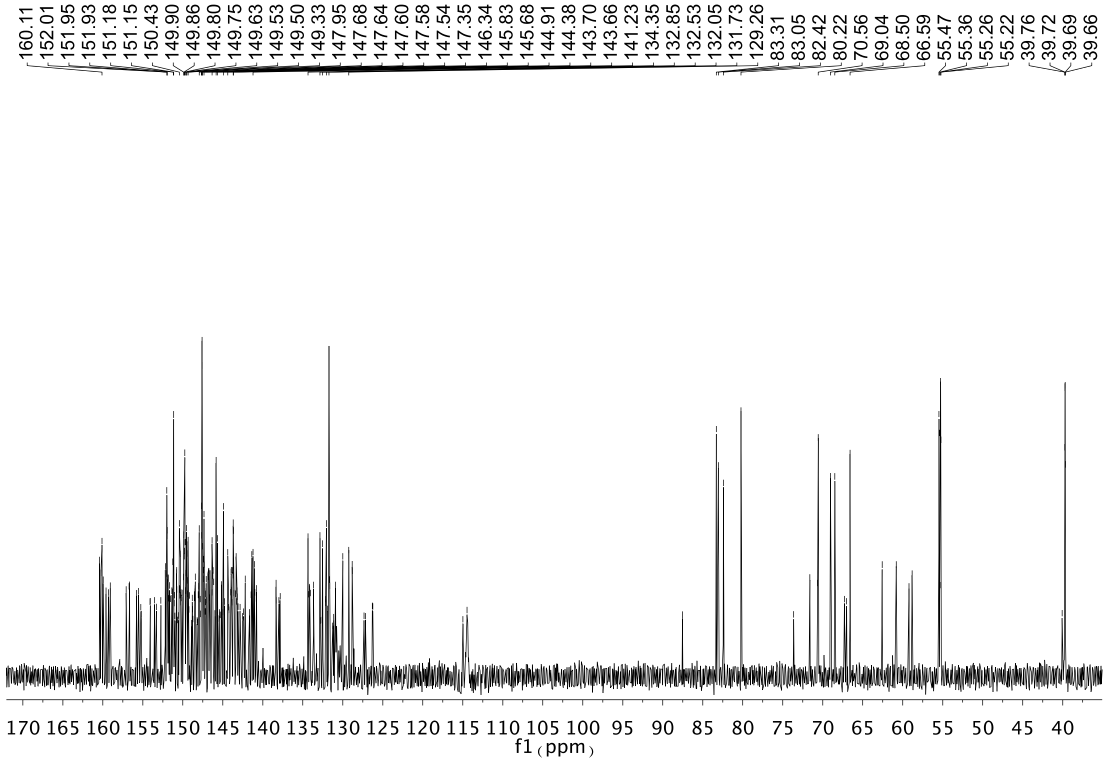

Figure S17. ^{13}C NMR spectrum of compound **70c** in a mixture of CS_2 and acetone-d6.

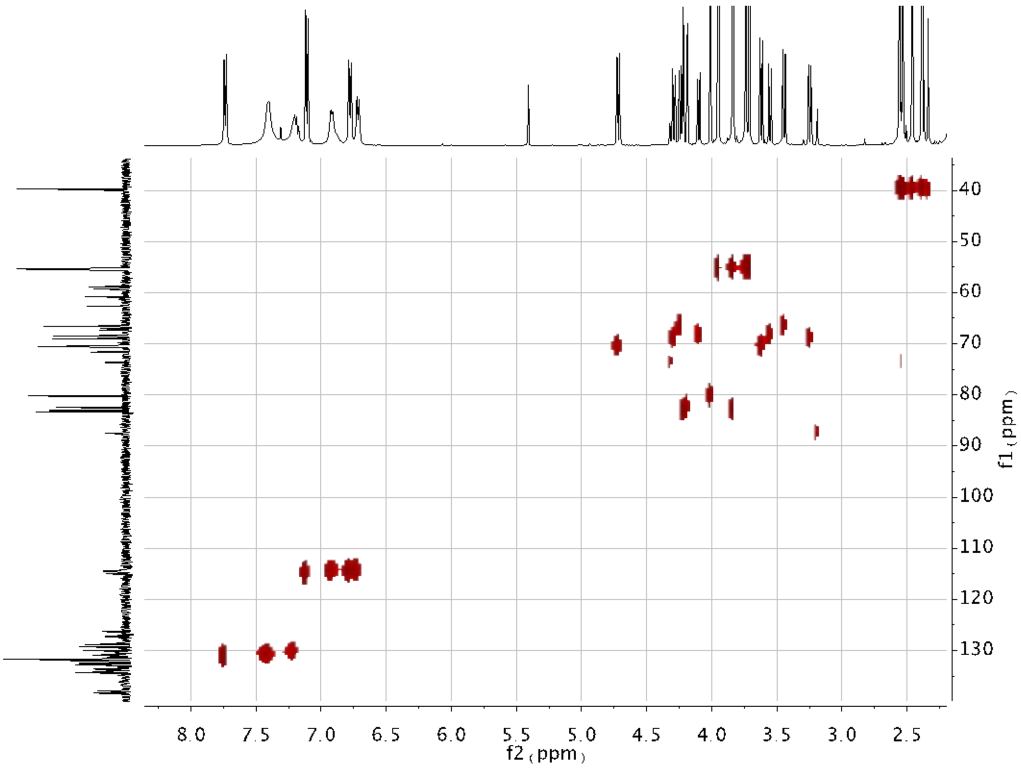

Figure S18. HSQC spectrum of compound **70c** in a mixture of CS_2 and acetone-d6.

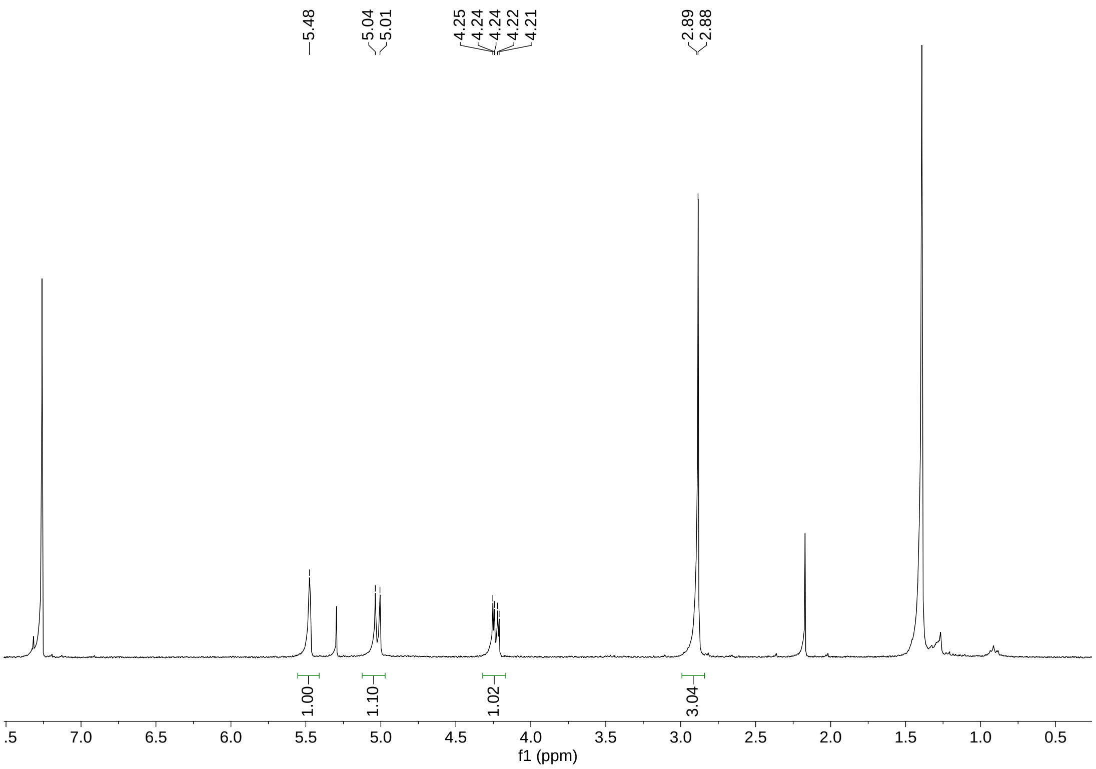

Figure S19. ^{1}H NMR spectrum of compound **60d** in a mixture of CS_2 and acetone-d6.

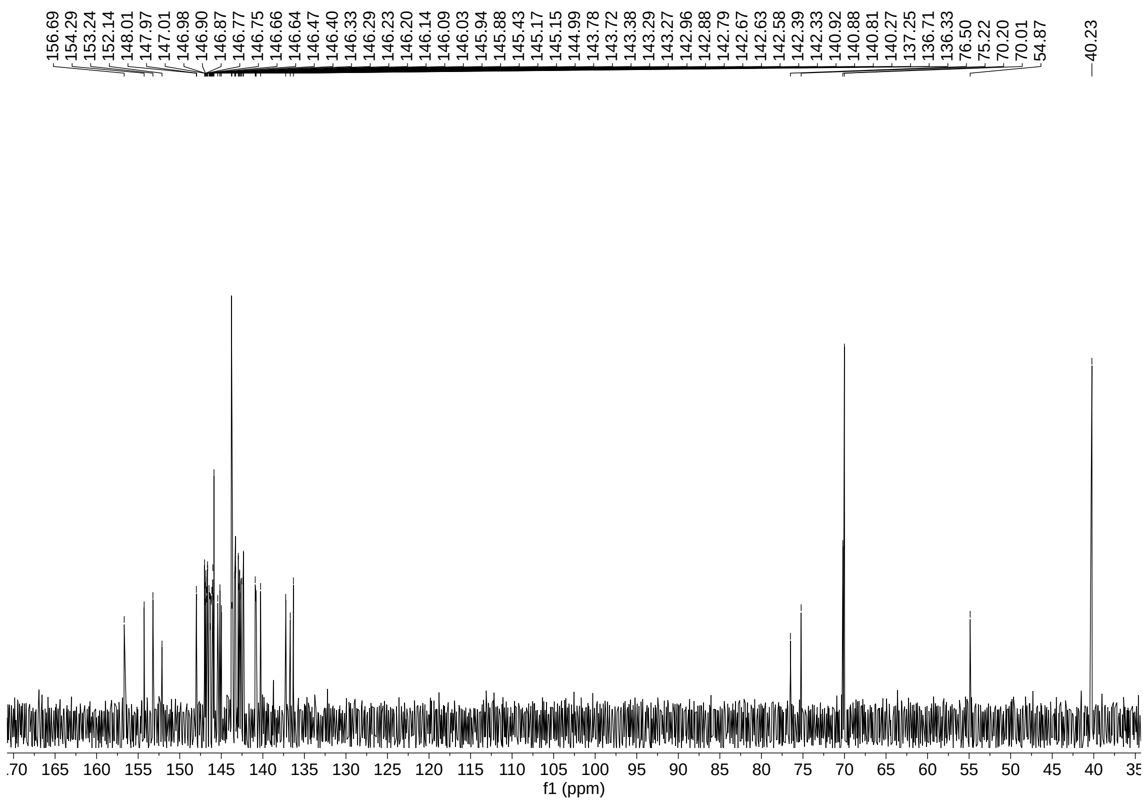

Figure S20. ^{13}C NMR spectrum of compound **60d** in a mixture of CS_2 and acetone-d6.

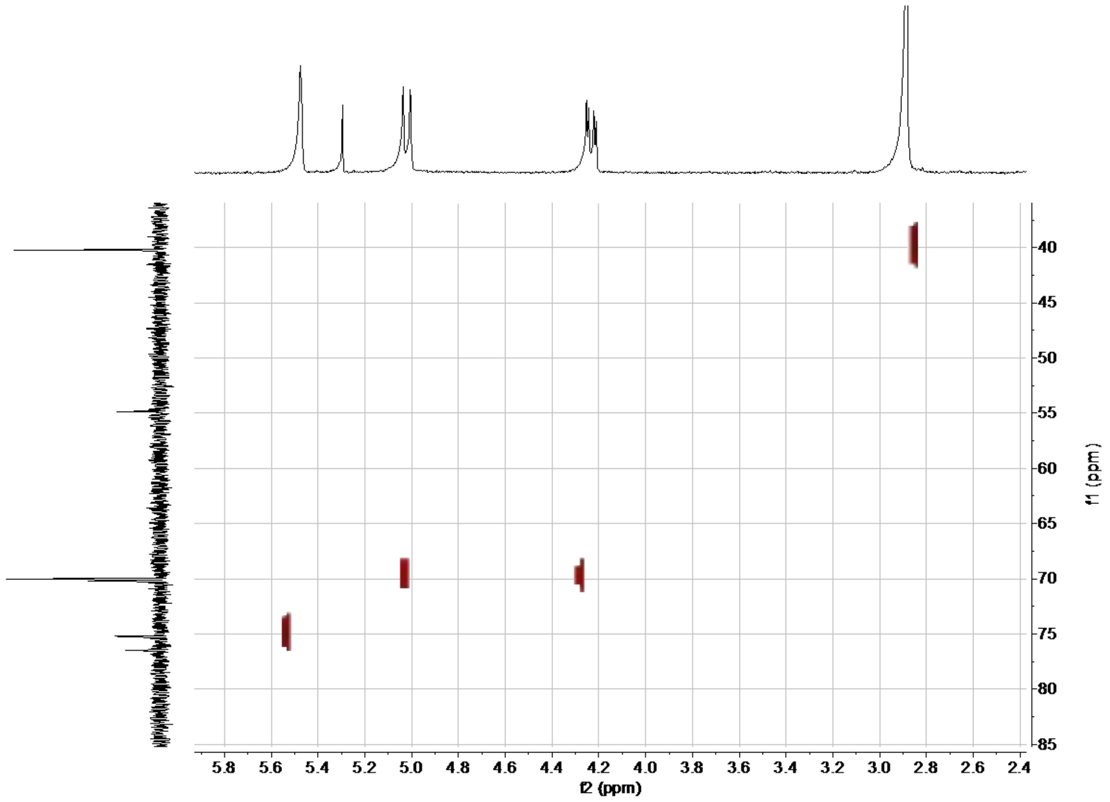

Figure S21. HSQC spectrum of compound **60d** in a mixture of CS_2 and acetone-d6.

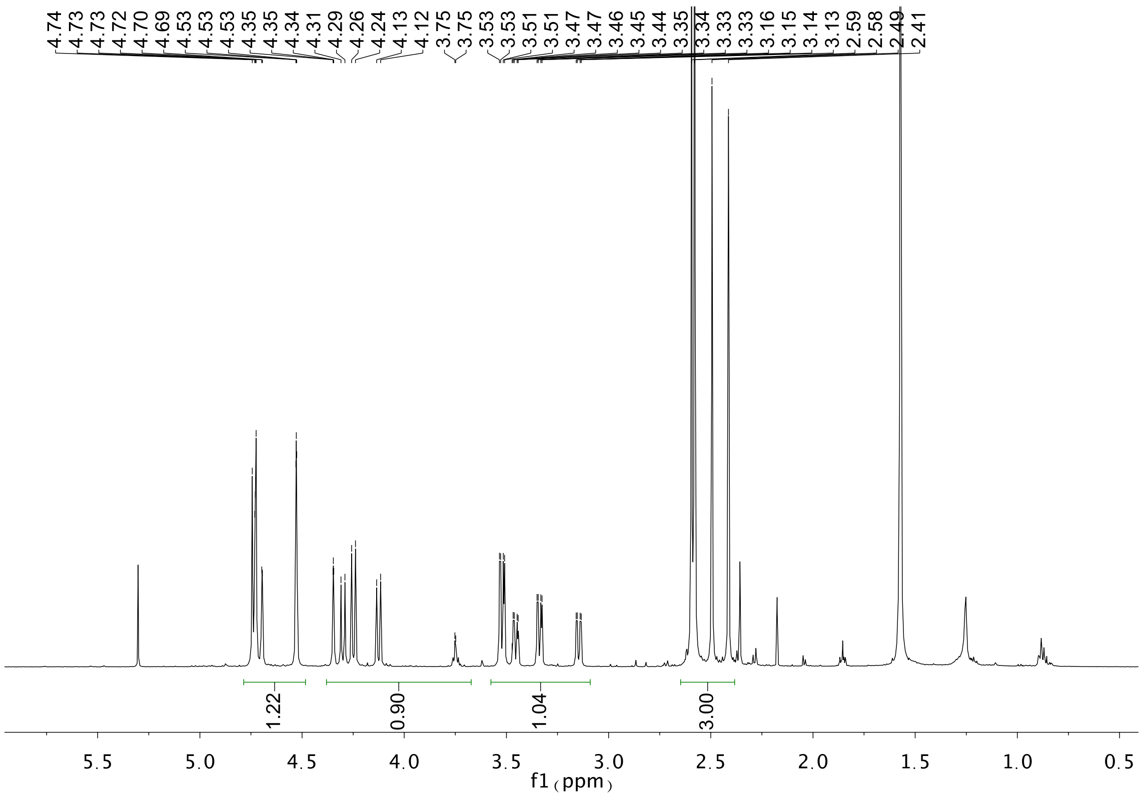

Figure S22. ^{1}H NMR spectrum of compound **70d** in a mixture of CS_2 and acetone-d6.

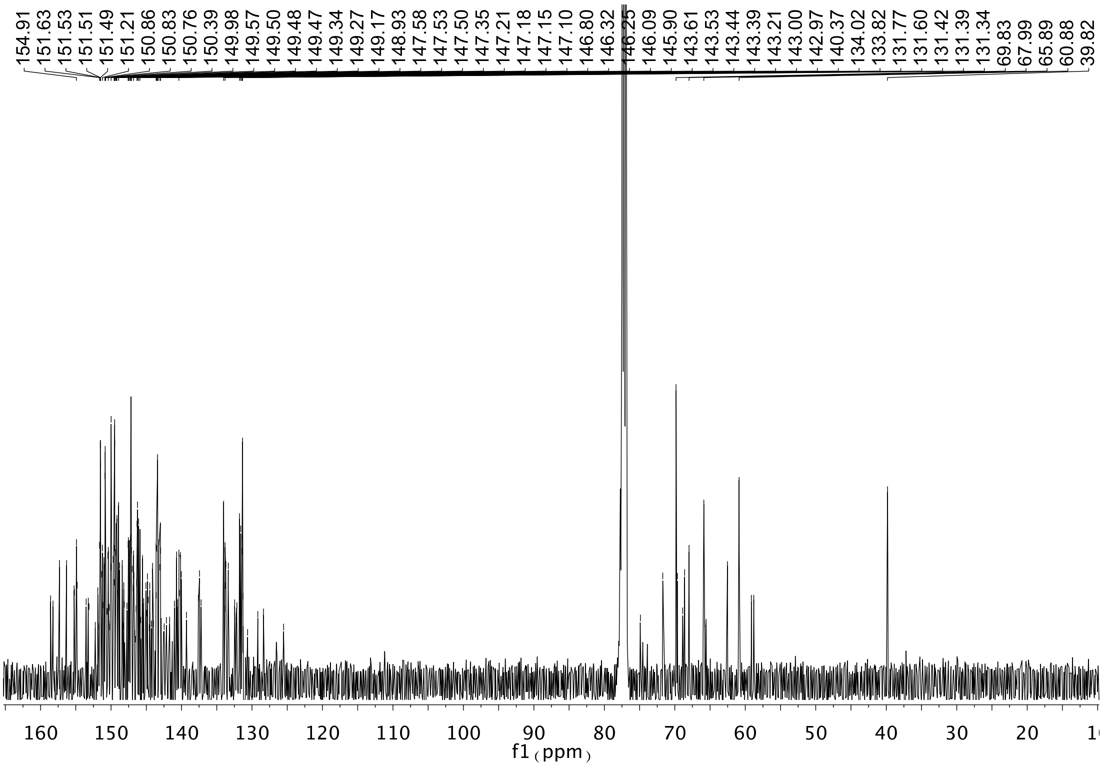

Figure S23. ^{13}C NMR spectrum of compound **70d** in a mixture of CS_2 and acetone-d6.

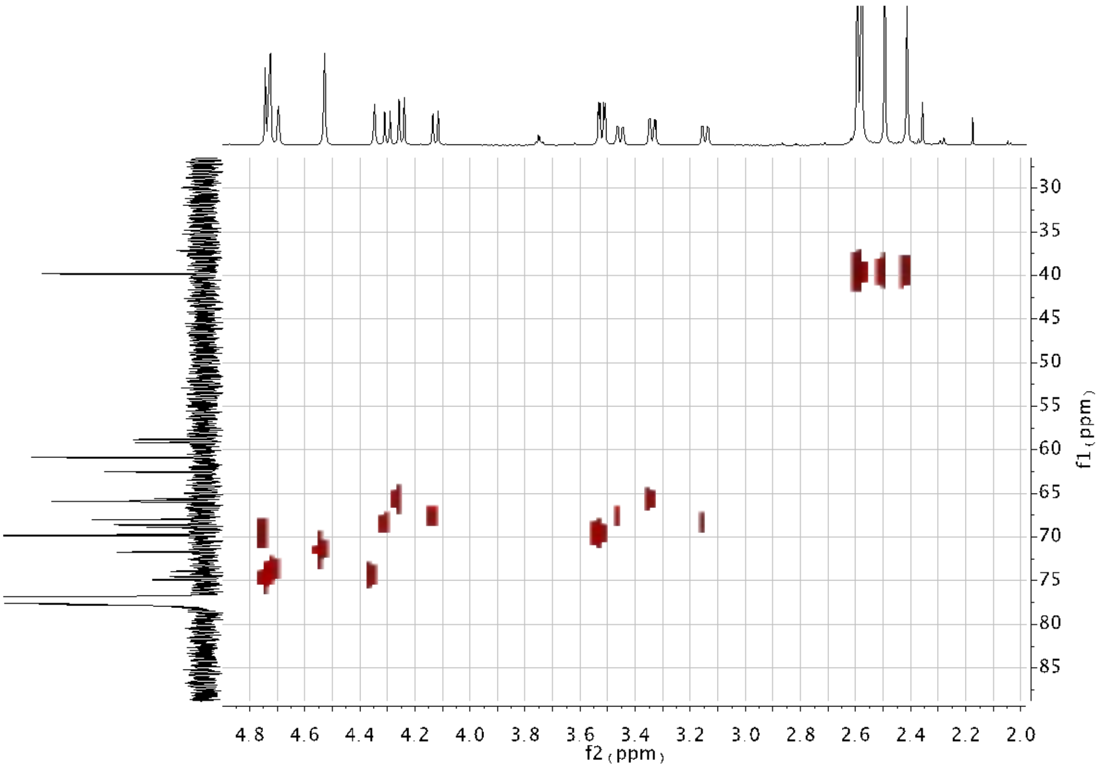

Figure S24. HSQC spectrum of compound **70d** in a mixture of CS2 and acetone-d6.

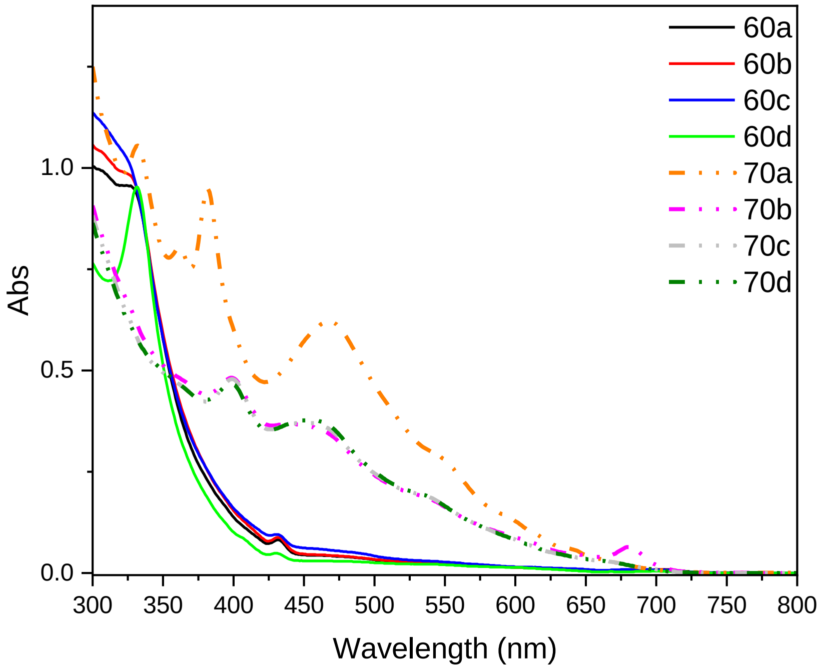

Figure S25. UV-Vis spectroscopy of the fullerenes in 1,2-dichlorobenzene.

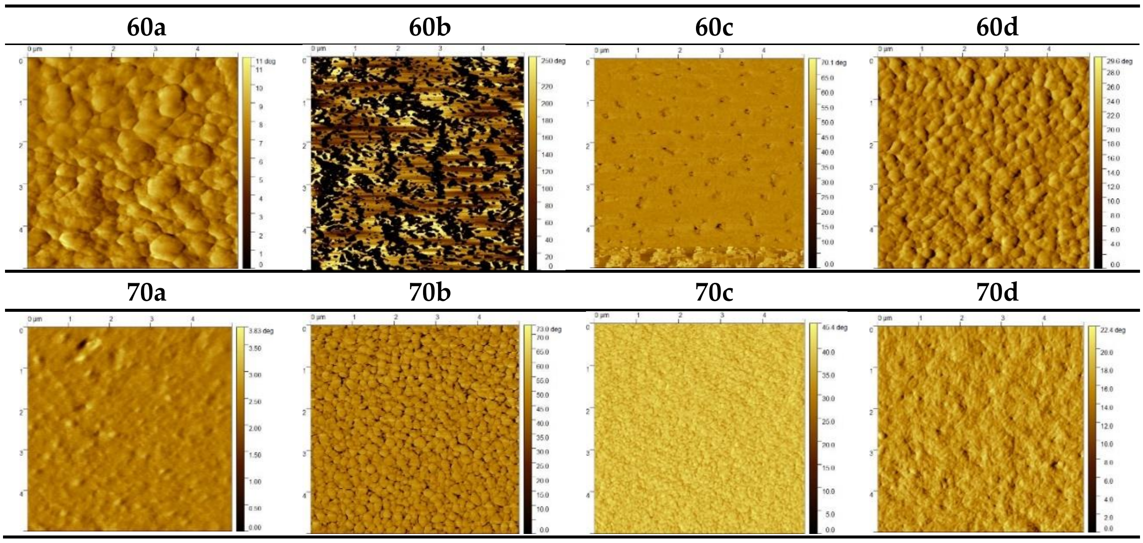

Figure S26. AFM phase images of PffBT4T-2OD based bulk-heterojunction films with the different fullerenes.

© 2019 by the authors. Submitted for possible open access publication under the terms and conditions of the Creative Commons Attribution (CC BY) license (http://creativecommons.org/licenses/by/4.0/).
